# The gendered nature of Muslim and Christian stereotypes in the United States

**DOI:** 10.1177/13684302221138036

**Published:** 2022-12-12

**Authors:** Caroline A. Erentzen, Veronica N. Z. Bergstrom, Norman Zeng, Alison L. Chasteen

**Affiliations:** 1 Toronto Metropolitan University, Canada; 2 University of Toronto, Canada

**Keywords:** Christian, gender, intersectional invisibility, intersectionality, Muslim, religious stereotypes

## Abstract

Despite the increasing diversity of religious affiliations in the United States, little research has explored the nature and structure of religious stereotypes of Muslims in America. The present research explores the gendered dimensions of stereotypes of both Muslims and Christians, using a multimethod approach. In Study 1, participants engaged in visual representations of intersectional and superordinate identities using Venn diagrams and slider tasks. Study 2 elicited open trait listings for religious, gender, and intersectional groups, with the most common traits reported for each group. In a conceptual replication, Study 3 asked participants to rate each group for the applicability of the most common traits identified in Study 2. Across the three studies, we found clear and consistent support for intersectionality effects. Unique stereotypic traits were identified for each intersectional group that were not present in either religious or gender superordinate identity. Stereotypes of Christians as a superordinate group contained a balanced representation of Christian men and Christian women traits. In contrast, Muslim stereotypes were strongly influenced by androcentric assumptions, with approximately 80% of the traits ascribed to Muslims overlapping with those of Muslim men. In addition, Muslim women were rated as significantly different from both Muslims and Muslim men on all trait evaluations. This was not observed with Christians, who showed little differentiation by gender. This research provides a rare systematic analysis of the gendered nature of religious stereotypes of Christians and Muslims and contributes to the developing literature on intersectionality and prototypicality.

## The Gendered Nature of Muslim and Christian Stereotypes in the United States

In January 2019, Ilhan Omar and Rashida Tlaib made history as the first Muslim women appointed to the United States House of Representatives, confronting many stereotypic assumptions about the passivity and oppression of Muslim women. While this victory was largely celebrated, Ilhan Omar in particular was accosted with accusations of being a “jihadi” with ties to “terrorist groups” (Anapol, 2018) and having “undisguised contempt” for America (Bryant, 2019). Not long before their election, former President Donald Trump enacted a controversial Muslim ban to restrict immigration and travel from seven predominantly Muslim countries. This was due, in large part, to his belief that Muslim men are violent, terror-associated, and threatening to the American way of life (Beydoun, 2017). The enactment of this ban led to an increase in violence toward Muslim Americans and the destruction of their religious properties and places of worship (e.g., Council on American–Islamic Relations, 2018). Such negative media coverage in the news and popular culture disproportionately depicts Muslims as violent extremists (Basit, 2018), belies the true diversity of the faith, and obscures the typically peaceful expressions of Islam worldwide (Bilge, 2010; Ismael & Measor, 2003).

This reflects an inherent tension and inconsistency in how Muslims are viewed in America. On the one hand, North American nations cherish religious freedom, acknowledging the diversity of religious beliefs that naturally exist in a multicultural society (e.g., Perry, 2015). On the other hand, religious minorities are often viewed with skepticism and distrust, due in large part to negative stereotypes that can lead to overt prejudice and discrimination (e.g., Crandall et al., 2011). As immigration patterns shift and evolve, Islam has emerged as the fastest growing faith in the United States. Estimates predict that, by the year 2040, Muslims will be the second largest religious group in America after Christians (Willingham, 2018). Our understanding of intergroup attitudes and beliefs becomes increasingly important as we move toward a more diverse cultural and religious mosaic. As we discover more about religious stereotypes of Muslims, we see that there may be different traits ascribed to Muslim men versus Muslim women. The purpose of the present research was to examine the content and structure of religious stereotypes toward Muslims, how they might compare to stereotypes of the largest religious group in America today (Christians), and the possible intersectional and gendered nature of these stereotypes.

## Stereotypes of Christians and Muslims

Some research has attempted to explore the nature and content of religious stereotypes of Christians. For example, Rios et al. (2015) had Christian and non-Christian participants numerically rate four religious groups (Muslims, Christians, atheists, and Jews) for warmth, competence, and trust in science. The results indicated an ingroup bias, with Christians finding their own religion members to be warmer and more competent than those of other faiths; non-Christians believed Christians to be less competent and trusting in science. More specific trait attributions were explored by Grove et al. (2020), who asked participants to evaluate atheists and Christians numerically on a list of 124 traits, finding that most of the Christian sample believed that Christians were religious, loving, charitable, moral, loyal to family, conservative, generous, passionate, proud, and peaceful. A second study by Grove et al. (2020) incorporated a participant sample with both Christians and non-Christians (atheists and agnostics), asking them to select five traits (from a longer list) that they thought were most descriptive of Christians and atheists. These participants similarly evaluated Christians as conservative, tradition loving, moral, closed-minded, and conventional.

A more complex and dimensional evaluation of Christians was observed by Bergstrom et al. (2022), who had both Christian and non-Christian participants provide up to five traits most often used socially to describe Christians, atheists, and agnostics. Christians were described as kind, religious, charitable, and conservative but also hypocritical, judgmental, intolerant, and pushy, indicating both positive and negative dimensions to Christian stereotypes. In a second study, Bergstrom et al. (2022) asked participants to rate the same groups numerically on a list of traits, finding that Christians were rated higher than atheists and agnostics as being moral, trustworthy, safe, patriotic, and predictable.

In comparison, Muslims have been consistently negatively evaluated. Zafar and Ross (2015) asked participants of Christian and non-Christian religious, and non-religious identities to list up to 10 traits and 10 emotional reactions they had in relation to several religious groups (Muslim, Christian, Sikh, Jewish, Hindu). Participants also completed a feeling thermometer and an Attitudes Toward Religious Groups Scale, assessing overall favorability toward that group. The authors found that Muslims were viewed the least warmly of the religious groups they assessed on a feeling thermometer. Muslims were described with the terms bad, strict, rude, terrorist, religious, and pray and most often evoked feelings including uncomfortable, neutral, happy, confused, and sad. In comparison, the most common traits for Christians in that study included good, loving, honest, caring, and faithful; the most common emotional reactions to Christians included happy, love, safe, care, and trust.

Other research has found that North Americans generally view Muslims more negatively than other religious groups (e.g., Putnam & Campbell, 2010), and that Muslims are thought of as oppressors of women, violent, radicalized, and cruel (Cainkar, 2004; Gottschalk & Greenberg, 2008). Edgell et al. (2006) conducted a telephone survey as part of a larger study on social and diversity attitudes, and asked participants about their reactions to a variety of racial, religious, and other groups. More than one quarter of respondents felt that Muslims did not represent their vision of American society, and one third would disapprove of their child marrying a Muslim person. Overall, nearly 80% of respondents did not approve of Muslims being in America to some extent. This may be related to blatant dehumanization processes, as observed by Lajevardi and Oskooii (2017), who presented participants with Kteily et al.’s (2015) Ascent of Man Scale. This scale presents images of different stages of human evolution from ape-like forms to modern-day upright humans; participants are asked to select where on the evolutionary scale they believe a particular group to be. Participants in Lajevardi and Oskooii’s (2017) study also completed a Muslim American Resentment Scale, which assesses a sense that Muslims do not belong in the United States, should not be trusted, and pose a threat to Americans. Participants rated White Americans as the most evolved group in America, whereas Muslims were considered the least evolved. Those who dehumanized Muslims also showed higher scores on the Muslim American Resentment Scale and greater support for laws that would increase surveillance and exclusion of Muslims from the United States.

## Superordinate Identities and Identity Assumptions

As outlined before, it appears that there are many stereotypes associated with religious identities. These groups have been studied primarily in their superordinate form, considering attitudes toward Christians or Muslims as an aggregate identity. There is, however, considerable diversity within each large category, much of which may be informed by other social identities. More specifically, perceivers often make assumptions about other nonspecified social identities when thinking about social groups, much of which may be based on privilege or dominant status in a social hierarchy. For example, when individuals think about Christians, they may be imagining those Christians as comprised of a particular ethnicity, gender, or nationality. Pursuant to a social identity dominance approach, some social identities tend to take dominance over others (e.g., Urban & Miller, 1998), suggesting that intersectional stereotypes are influenced more strongly by those identities. Lorde (1995) refers to the “mythical norm” in our society, normally “defined as white, thin, male, young, heterosexual, Christian and financially secure” (p. 192). Expanding on these concepts, Purdie-Vaughns and Eibach (2008) presented an intersectional invisibility hypothesis in which certain identities are deemed prototypical of various groups; specifically, that male, White, and heterosexual are the default identities. As a person has more and more nonprototypical identities (e.g., Black queer women), they will experience intersectional invisibility, being overlooked or forgotten.

Ghavami and Peplau (2013) found that stereotypes of White people overlapped more with stereotypes of White men than with stereotypes of White women, supportive of the notion that men are viewed as the default gender. Moreover, stereotypes of women overlapped more with stereotypes of White women than with stereotypes of Black women, supportive of the notion that White ethnicity is the perceived default ethnicity in America. Eagly and Kite (1987) assessed stereotypes of nationality and gender, finding that stereotypes of various nations were more similar to stereotypes of male members of that nation than of female members of that nation. This work indicates that, when studying superordinate identity categories such as religion, one must consider the possibility that gender identities are informing these stereotypes. In particular, male identity may be assumed as a default if female identity is not specifically named. The present research sought to study superordinate religious identities as well as those informed by the specific intersection of both religion and gender.

## An Intersectional Approach

As noted, research on stereotyping has historically focused on single identities, considering only ethnicity or gender or religion. As our knowledge and understanding of stereotype structure and content evolved, a more nuanced approach emerged with Crenshaw’s (1991) foundational work on intersectionality. Pursuant to this approach, it is not enough to consider stereotyping and prejudice that might apply to a unitary social identity; rather, we must consider the interplay between multiple social identities. That is, rather than speaking of attitudes toward Muslim persons, for example, we must consider the different experiences of Muslim men as compared to Muslim women. Thus, multiple social identities create unique experiences of prejudice, which cannot be reduced to the sum of their individual parts. A large body of empirical work has been developed in support of this perspective, demonstrating that a unitary approach to identity and stereotyping is no longer sustainable (e.g., Kang & Bodenhausen, 2015). Rather, the way we think about a social group must be considered through the lens of the other group identities involved (Ghavami & Peplau, 2013).

Whereas little research has explored the intersectional stereotypes of gender and religious identities, there is abundant work exploring the interplay of gender, race, and sexual orientation. One key approach emerging from this literature is the double jeopardy hypothesis, which explains that having multiple stigmatized identities increases one’s experience of disadvantage (e.g., Reid, 1984). For example, Black women would experience more prejudice than Black men by virtue of their disadvantaged race and gender. Crenshaw (1991) proposed a multiplicative approach, suggesting that social inequality is magnified significantly as a greater number of stigmatized identities are present. That is, the experience of being Black and a woman is more marginalizing than the simple additive effects of being Black and a woman separately.

Building on these concepts, we might consider that the experience of discrimination will be unique to each intersectional identity rather than the sum of the individual identities (e.g., Peplau et al., 1999; Remedios & Snyder, 2015). Further, there may be unique stereotypic traits ascribed to a Black woman, for example, that are not found in the general categories “Black” or “woman.” Support for this approach is accumulating. For example, Ghavami and Peplau (2013) asked participants to list, in an open-ended format, five traits associated with randomly assigned groups that varied by ethnicity, gender, or intersectional identity. Unique traits were generated for intersectional identities that were not present in either superordinate group. Black men were described as “rappers” and “quick to anger,” whereas Black women were described as “confident” and “assertive,” but these traits were not present in the superordinate stereotypes of Black people or men/women more generally. Similarly, Preddie and Biernat (2020) found that gay Black male stereotypes included unique traits (i.e., outgoing, dramatic) not found in the stereotypes of men, gay men, or Black men. Might a similar process apply to Muslim women and Muslim men stereotypes?

### Gendered stereotypes of Muslims

Interestingly, the existing literature exploring attitudes toward Muslims has intuitively incorporated a gendered element. Whereas Muslim men are often viewed as aggressive, hot tempered, and intolerant (Gottschalk & Greenberg, 2008; Perry, 2014; Sides & Gross, 2013), Muslim women are often seen as oppressed and subjugated due to their religious head coverings (Chakraborti & Zempi, 2012; Perry, 2014; Zempi & Chakraborti, 2014). Bilge (2010) notes the inherent tension in attitudes toward Muslim women, with many North Americans seeing the Muslim headscarf as a “symbol of submission” to men and simultaneously as a “symbol of resistance” to the West (Bilge, 2010, p. 14). Although the gendered aspects of Muslim stereotypes have been proposed in the literature (e.g., Chakraborti & Zempi, 2012; Perry, 2014; Sides & Gross, 2013; Zempi & Chakraborti, 2014), there is limited research undertaking a detailed, granular-level analysis of the content of stereotypes associated with these groups, and very little research exploring the possible gendered dimension of Christian stereotypes.

## The Present Research

The goal of the present research was to use an exploratory and descriptive approach to understand the content of gendered religious stereotypes of Muslims and Christians in the United States of America. As a first step, Study 1 explored the general perceived similarity between groups using visuospatial representations of group similarity. Study 2 elicited open-ended stereotype content from participants for Christians, Muslims, men, women, and their intersectional identities. The most common traits generated for each group were identified and compared to determine how many traits overlapped between superordinate and intersectional identities. Finally, Study 3 conceptually replicated Study 2 by presenting participants with a series of the most common stereotypic traits elicited in Study 2 and asking them to rate groups numerically on each trait. Participants in Study 3 were also asked to report any identity assumptions they had made about each group in terms of its ethnicity, immigration status, and gender (for religious superordinate groups), to explore the possible invisibility of identities and whether religious stereotypes are driven more by male, female, or nongendered assumptions. Although exploratory in nature, we propose a series of hypotheses for both superordinate and intersectional identities that are derived from the default assumptions often made about identities in North America.

### Hypotheses

#### Superordinate religious identities

Religious superordinate identities will be associated more closely with male representations of the faith due to androcentric assumptions about the default identity in North America (i.e., that, absent further information, the default person is White, male, heterosexual, younger, and otherwise taking the privileged form of any identity).

#### Superordinate gender identities

Gender categories will be seen as more similar to Christianity than to Islam, as Christianity is the dominant religious identity in North America.

#### Intersectional identities

Intersectional identities will be considered more similar to their religious superordinate identity than to their gender superordinate identity, as religion is a smaller and likely more informative category than gender. For example, Muslim women will be considered more similar to Muslims than to women, as being an adherent to the Muslim faith is a numerically smaller category than being a woman.

## Study 1

The purpose of Study 1 was to explore the perceived similarity between intersectional groups and their superordinate identities through visuospatial representation. As noted before, it was hypothesized that religious identities would be seen as more similar to male concepts than to female concepts, that gender identities would be seen as more similar to Christian concepts than to Muslim concepts, and that intersectional identities would be considered more similar to their religious superordinate identity than to their gender superordinate identity. This project was preregistered on the Open Science Framework (https://osf.io/hrkmg/?view_only=c36bfca0d09f4034a1ea40d6f695ab7d).

### Participants

A power analysis was performed with G*Power (Version 3.1.9.7), specifying a small effect size (*d* = 0.18) and power of .80, and incorporating two-tailed paired samples *t* tests, which resulted in a recommended sample of 245 participants. To offset potential data loss, 261 participants were recruited from Amazon Mechanical Turk in exchange for monetary compensation (US$1.30). All participants resided within the United States, verified by their location latitude and longitude. Participants who failed to complete the study (*n* = 3) or who showed inattentive responding through failed attention checks (*n* = 13) were excluded from analyses, resulting in a final sample size of 245 participants. Demographic information for this sample is provided in Table 1.

**Table 1.** Demographic characteristics of participants across studies

	Study 1		Study 2		Study 3	
	*N*	%	*N*	%	*N*	%
**Ethnicity**						
White	192	78.37	200	76.63	233	75.65
Black	18	7.35	36	13.79	41	13.31
Hispanic	6	2.45	8	3.07	12	3.90
East/Southeast Asian	12	4.90	6	2.30	10	3.25
South Asian	1	0.41	5	1.92	2	0.65
Multiracial/other ethnicity	16	6.53	6	2.30	10	3.25
**Religion**						
Christian/Catholic	118	48.16	167	63.98	158	51.30
Atheist	53	21.63	37	14.18	48	15.58
Agnostic	45	18.37	34	13.03	76	24.68
Jewish	1	0.41	4	1.53	3	0.97
Buddhist	2	0.82	4	1.53	1	0.32
Hindu	1	0.41	3	1.15	2	0.65
Muslim	4	1.63	1	0.38	1	0.32
Other faith/multifaith	21	8.57	11	4.21	19	6.17
**Sexual orientation**						
Heterosexual	216	88.16	195	74.71	266	86.36
Gay/lesbian	6	2.45	4	1.53	6	1.95
Bisexual	23	9.39	54	20.69	33	10.71
Other orientation	0	0.00	5	1.92	3	0.97
Declined answer	0	0.00	3	1.15	0	0.00
**Education level**						
PhD or professional degree (MD, JD)	3	1.22	1	0.38	4	1.30
Master’s degree	30	12.24	60	22.99	50	16.23
Bachelor’s degree	122	49.80	130	49.81	137	44.48
College diploma	22	8.98	17	6.51	34	11.04
Some college/ university	41	16.73	31	11.88	47	15.26
High school diploma	26	10.61	19	7.28	36	11.69
Less than high school	1	0.41	0	0.00	0	0.00
Declined answer	0	0.00	3	1.15	0	0.00
**Individual differences**	***M***	***SD***	***M***	***SD***	***M***	***SD***
Dawkins Religiosity Scale^1^	4.75	2.28	5.08	2.11	4.47	2.42
Political orientation^2^	3.66	1.89	4.11	1.99	3.67	1.82

*Note*. ^1^Participants completed an adapted version of the Dawkins Spectrum of Theistic Probability Scale (Dawkins, 2006), a single-item measure assessing belief in a monotheistic god (1 = *I am 100% certain God does not exist*, 7 = *I am 100% certain God* exists). ^2^Participants were also asked to report their political orientation on a single item (1 = *extremely liberal*, 7 = *extremely conservative*).

MD = Doctor of Medicine, JD = Juris Doctorate (law degree).

### Materials

#### Venn diagrams

**Figure 1.**
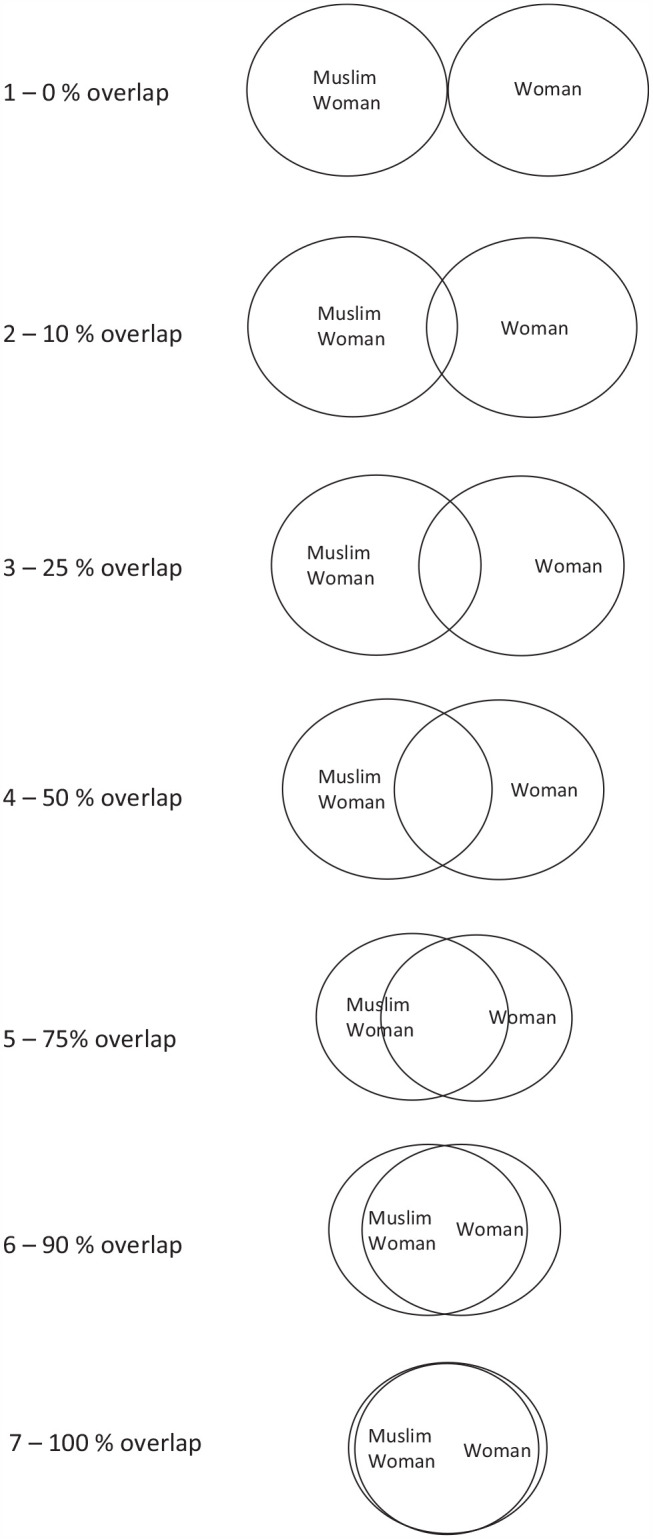
Example of Venn diagrams depicting visual overlap between Muslim women and women. *Note*. Circles which are farther apart (e.g., closer to 1) indicate that the participant thinks of the two categories as distinct from each other. Here, a score of 1 would suggest that the participant does not see any similarity at all between Muslim women and the general category of women. Circles which are very close together indicate that the participant thought of the two identities as very similar. Here, a score of 7 would suggest that the participant considers Muslim women and women to be the same essential identity.

Participants were presented with a series of Venn diagrams, visually representing two circles moving in increasing degrees of proximity and overlap. This scale was adapted from similar measures prepared by Aron et al. (1991, 1992), which measured perceived similarity between the self and others, and by Tropp and Wright (2001), which measured perceived overlap between self and ingroup concepts (see also Wright et al., 2003). This procedure has been adapted recently for comparisons of group similarity by Preddie and Biernat (2020), who compared perceived similarity of intersectional male groups on the basis of race and sexual orientation. In the present study, we wanted to explore conceptual overlap between intersectional and superordinate group identities. Participants were assigned two nonoverlapping intersectional identities to evaluate separately (e.g., Muslim women and Christian men) with the Venn diagram task. All participants evaluated the overlap between religious and gender superordinate groups (Muslim and men, Muslim and women, Christian and men, Christian and women), as depicted in Figure 1.

Participants were given the following instruction, “Looking at the images below, select the image that best represents the degree to which you think the concept ‘Muslim’ overlaps with the concept ‘Woman.’” They would also report their perceived overlap between the concepts “Muslim and man,” “Christian and woman,” “Christian and man,” “man and woman,” and “Muslim and Christian.” The presentation of each identity on the left or right was counterbalanced, and the order in which the superordinate groups were presented was randomized. For each pair of superordinate identities, participants selected the circles that best represented their perceived overlap between the two groups, with response options including 1 (0% overlap), 2 (10% overlap), 3 (25% overlap), 4 (50% overlap), 5 (75% overlap), 6 (90% overlap), and 7 (100% overlap). This 7-point scale is consistent with that employed in the Venn diagram tasks described by Aron et al. (1992) and Wright et al. (2003), in which the lower end of the scale (1) represents 0% overlap between circles, the midpoint of the scale (4) represents 50% overlap, the high end of the scale (7) represents 100% overlap of the circles, and the remaining circles represent intermediate overlap.

In addition, participants were randomly assigned to review two nonoverlapping intersectional identities for the perceived overlap between that intersectional identity and its superordinate groups. For example, a participant assigned to evaluate Muslim women would be asked to report the perceived overlap between Muslim women and women, and the perceived overlap between Muslim women and Muslims. They would then proceed to make a similar evaluation for Christian men, reporting that group’s overlap with Christians and with men.

#### Sliders

**Figure 2.**
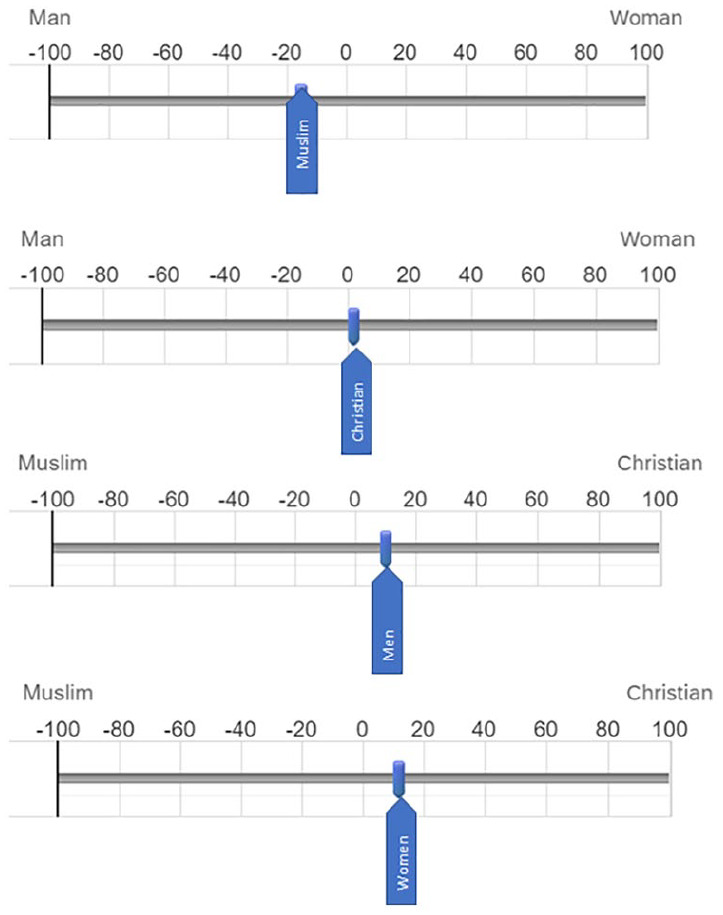
Mean slider ratings for perceived similarity between religious and gender identities. *Note*. The blue slider represents a superordinate identity. Participants were asked to move the slider toward one end of the scale or the other, with the anchors representing two competing variations of another superordinate identity. For example, if the sliders represent a religious superordinate identity (e.g., Muslim), participants would have to choose whether to move the Muslim slider closer to the man end of the scale or to the woman side of the scale, reflecting their perception of Muslims as being more masculine or feminine in nature. Participants could also leave the slider in place if they did not believe Muslims (in this example) were more similar to male or female identities. *Note*. Please refer to the online version of the article to view this figure in colour.

For each superordinate group, participants were given a forced-choice option. For example, they were told, “The slider in the middle represents the concept ‘Christian.’ Drag the slider in the direction you think Christian is most similar.” At each end of the slider was a gender, and the participant was instructed to move the slider (i.e., Christians) toward the left (men) or toward the right (women). The placement of gender identities on the scale was counterbalanced. Similarly, negative scores represented Muslim religion and positive scores represented Christian religion for the gender sliders. Smaller numbers closer to zero suggest no gendered concepts for religious sliders, and no religious concepts pulling the gender sliders. Responses were reverse-coded where necessary such that negative scores reflected male gender or Muslim religion, and positive scores reflected female gender or Christian religion. See Figure 2 for a visual representation of the slider task.

### Procedure

Participants were randomly assigned to review two nonoverlapping intersectional identities (e.g., Muslim men and Christian women) for similarity to their respective superordinate identities on the sliders and Venn diagrams. Following completion of these tasks, participants provided demographic information and were debriefed.

### Data Analysis Plan

As a first step, we compared the perceived similarity of superordinate groups to each other on the Venn diagram task, followed by the perceived overlap between intersectional identities and each of their superordinate identities. To do so, two paired sample *t* tests were conducted comparing the mean overlap rating of the religious superordinate groups to each gender superordinate group (i.e., Muslims and men vs. Muslims and women; Christians and men vs. Christians and women). In addition, we compared the degree of perceived overlap between each intersectional identity and its two superordinate identities. This resulted in an additional four paired *t* tests (i.e., Muslim men and Muslims vs. Muslim men and men; Muslim women and Muslims vs. Muslim women and women; Christian men and Christians vs. Christian men and men; Christian women and Christians vs. Christian women and women). In total, six pairwise *t* tests were conducted on the Venn diagram tasks. A Bonferroni error correction was applied to reduce the risk of Type I error, resulting in a significance threshold of *p* < .008.

This general comparison of mean differences was followed by an examination of the slider ratings as against a neutral midpoint rating of zero. That is, if religious identities are seen as relatively gender-neutral, the mean slider score would not differ significantly from zero, not having moved toward the male or female anchor. Four pairwise comparisons were made for the slider tasks (i.e., Christian vs. men/women; Muslim vs. men/women; men vs. Muslim/Christian; women vs. Muslim/Christian), and a Bonferroni error correction of *p* < .0125 was applied.

### Results

#### Venn diagram similarity ratings

##### Superordinate groups

Paired sample *t* tests were conducted to explore the potential gendered nature of religious stereotypes. Muslims were considered more similar to men (*M* = 3.10, *SD* = 1.66) than to women (*M* = 2.86, *SD* = 1.47), *t*(244) = −3.66, *p* < .001, *d* = 0.23. With respect to the subjective similarity ratings, a rating of 3.00 represents 25% perceived overlap between categories. When we consider the standard deviations, Muslims were considered to have between 10 and 75% similarity to the category men, whereas they were considered to overlap roughly 10 to 50% with the category women. Contrary to hypotheses, Christians were not considered more similar to men (*M* = 3.34, *SD* = 1.67) versus women (*M* = 3.32, *SD* = 1.64), *t*(244) = −0.39, *p* = .698, *d* = 0.03. With reference to the subjective overlap of these ratings, participants believed that Christians overlapped with men and with women slightly more than 25%, ranging from roughly 10 to 75% for each gender superordinate identity. This suggests that Muslim stereotypes may be informed by masculine assumptions, whereas Christian stereotypes may incorporate a more balanced gender representation.

##### Intersectional groups

Consistent with hypotheses, each intersectional group was considered to overlap more with its religious identity than with its gender identity. More specifically, Christian men were rated more similar to Christians (*M* = 5.68, *SD* = 1.61) than to men (*M* = 5.07, *SD* = 1.61), *t*(121) = 4.58, *p* < .001, *d* = 0.42. Factoring in standard deviations, this equates to approximately a perceived 50 to 100% overlap of Christian men with Christians, and an approximately 25 to 90% overlap for Christian men and men. Similarly, Christian women were considered more similar to Christians (*M* = 5.37, *SD* = 1.47) than to women (*M* = 4.02, *SD* = 1.78), *t*(123) = 8.04, *p* < .001, *d* = 0.72. When we factor in standard deviations, this equates to a perceived overlap of 50 to 100% between Christian women and Christians, and between 10 and 90% overlap between Christian women and women. Muslim men were considered more similar to Muslims (*M* = 5.27, *SD* = 1.73) than to men (*M* = 3.89, *SD* = 1.98), *t*(123) = 7.68, *p* < .001, *d* = 0.69. Considering standard deviations, Muslim men were thought to conceptually overlap with Muslims between 25 and 100%, and with the concept of men between 10 and 90%. Finally, Muslim women were rated more similar to Muslims (*M* = 5.68, *SD* = 1.45) than to women (*M* = 4.94, *SD* = 1.81), *t*(120) = 4.48, *p* < .001, *d* = 0.41. Considering standard deviations, Muslim women were thought to overlap conceptually with Muslims between 50 and 100%, and with women between 25 and 90%.

#### Slider similarity ratings

As seen in Figure 2, Muslims were more likely to be moved toward the male end of the sliding scale (*M* = −17.30, *SD =* 31.54), *t*(220) = −8.15, *p* < .001, *d* = 0.55. Christians, however, were not significantly more likely to be seen as male or female (*M* = 1.68, *SD* = 27.50), *t*(219) = 0.91, *p* = .367, *d* = 0.06, confirming the results observed with the Venn diagram task. A similar process was undertaken with gender, testing scores against a midpoint of zero. Consistent with hypotheses, men were deemed to be more similar to Christians than to Muslims (*M* = 7.60, *SD* = 28.60), *t*(220) = 3.95, *p* < .001, *d* = 0.27, and women were also deemed to be more similar to Christians than to Muslims (*M* = 12.25, *SD* = 32.08), *t*(220) = 5.65, *p* < .001, *d* = 0.38. This likely reflects assumptions about the greater probability of a Christian (vs. Muslim) affiliation for any given man or woman in the US.

### Discussion

The results of Study 1 reveal that, although all intersectional identities were considered more similar to their religious identity than to their gender identity, there is a gendered (and male) aspect to stereotypes of Muslims that does not exist for Christians. More specifically, Muslims as a superordinate group were considered conceptually closer to men than to women in the slider task, whereas Christians did not show any difference in proximity to male or female gender. Having established a potential gendered aspect of these religious stereotypes, Study 2 explored the content of intersectional religious stereotypes in more detail.

## Study 2

The goal of Study 2 was to identify the specific traits associated with each superordinate and intersectional identity, and to determine whether gender was more important in informing the stereotypes of Muslims versus Christians. This study was preregistered on the Open Science Framework (https://osf.io/je3ms/?view_only=98d6f1cac0ae4f3799f9bee923bc437d). As this research was exploratory in nature, we did not make firm hypotheses about the content of these stereotypes. However, pursuant to an intersectionality hypothesis, it was predicted that unique stereotypes would emerge for intersectional identities that are not present in the superordinate categories. For example, unique traits would be ascribed to Christian men that are distinct from those of Christians and of men.

### Method

#### Participants

An a priori power analysis was performed with G*Power (Version 3.1.9.7), specifying a small effect size (*f* = .10) and power of .80, resulting in a recommended sample of 240 participants (60 per block). To offset potential data loss, we oversampled for the study, recruiting 400 participants from Amazon Mechanical Turk’s online platform in exchange for monetary compensation (US$1.30). Participants were recruited from the United States of America, with residency verified by latitude and longitude metadata. After removing attempts at duplicated responses, nonsensical answers, and nonattentive responding, the final sample included 261 participants (156 men, 105 women; *M*_age_ = 35.45 years, *SD* = 10.22). Demographic characteristics are provided in Table 1.

#### Materials and procedure

Participants were randomly assigned to provide evaluations of three groups with nonoverlapping identities. This included one intersectional identity (e.g., Muslim man) and two superordinate identities that did not overlap with the intersectional identity assigned (e.g., women, Christian). For each group assigned to them, participants were asked to “list up to five words or short phrases that relate to stereotypes MOST PEOPLE hold about [group].” As seen in Tables 2a, 2b, and 2c, between 80 and 94 participants were randomly assigned to evaluate each group. After completing the traits generation tasks, participants provided demographic information and were debriefed.

**Table 2a.** Ten most commonly generated traits for Muslims, Muslim men, and Muslim women: Study 2.

Muslims (*n* = 85)				Muslim men (*n* = 91)				Muslim women (*n* = 84)			
	*N*	%	α		*N*	%	α		*N*	%	α
**Trait**				**Trait**				**Trait**			
Religious	28	32.94	.87	Terrorist	30	32.97	.97	Oppressed	33	39.29	.88
Terrorist	25	29.41	.99	Religious	28	30.77	.72	Submissive	25	29.76	.95
Violent	15	17.65	.83	Misogyny	25	27.47	.94	Religious	20	23.81	.86
Misogyny	13	15.29	.98	Strict	20	21.98	.93	Headscarf	13	15.48	.97
Prejudiced	13	15.29	.80	Radical	16	17.58	.67	Brave	13	15.48	.96
Aggressive	12	14.12	.82	Violent	15	16.48	.98	Attractive	13	15.48	.99
Headscarf	12	14.12	.96	Good	14	15.38	.97	Quiet	13	15.48	.94
Good	11	11.76	.99	Aggressive	13	14.29	.98	Meek	13	15.48	.93
Radical	10	11.76	.81	Bearded	12	13.19	.95	Mistreated	12	14.29	.76
Middle Eastern	9	10.59	.99	Middle Eastern	10	10.99	.99	Terrorist	10	11.90	.98

*Note*. If two traits were tied in tenth place, both are presented. Alphas represent intercoder reliability for that term.

**Table 2b.** Ten most commonly generated traits for Christians, Christian men, and Christian women: Study.

Christians (*n* = 88)				Christian men (*n* = 90)				Christian women (*n* = 90)			
	*N*	%	α		*N*	%	α		*N*	%	α
**Trait**				**Trait**				**Trait**			
Religious	31	35.72	.89	Religious	32	40.00	.93	Religious	30	33.33	.95
Prejudiced	19	21.59	.96	Good	18	22.50	.89	Nice	22	24.44	.98
Uptight	18	20.45	.89	Prejudiced	15	18.75	.64	Uptight	21	23.33	.80
Nice	14	15.91	.98	Misogyny	12	15.00	.98	Good	21	23.33	.91
Judgmental	13	14.77	.99	Honest	12	15.00	.96	Homemaker	20	22.22	.91
Good	12	13.63	.92	Brave	12	15.00	.99	Faithful	18	20.00	.93
Conservative	12	13.63	.99	Nice	11	13.75	.97	Pure	15	16.67	.95
Faithful	12	13.63	.69	Strict	10	12.50	.97	Attractive	11	12.22	.86
Hypocritical	11	12.50	.98	Conservative	10	12.50	.97	Submissive	11	12.22	.88
Christmas	10	11.36	.95	Faithful	9	11.25	.97	Self-righteous	10	11.11	.86
Church	10	11.36	.87	Attractive	9	11.25	.72	Conservative	10	11.11	.99

*Note*. If two traits were tied in tenth place, both are presented. Alphas represent intercoder reliability for that term.

**Table 2c.** Ten most commonly generated traits for men and women: Study 2.

Men (86 traits generated; *n* = 88)				Women (74 traits generated; *n* = 94)			
	*N*	%	α		*N*	%	α
**Trait**				**Trait**			
Strong	39	44.32	.95	Caring	27	28.72	.79
Unemotional	21	23.86	.92	Emotional	22	23.40	.93
Aggressive	21	23.86	.96	Nice	20	21.28	.97
Mean	17	19.32	.72	Weak	19	20.21	.89
Arrogant	12	13.64	.74	Attractive	17	18.09	.96
Brave	12	13.64	.80	Good	15	15.96	.99
Attractive	11	11.70	.94	Strong	14	14.89	.94
Controlling	10	11.36	.88	Brave	11	11.70	.97
Provider	10	11.36	.83	Unintelligent	9	9.57	.88
Masculine	10	11.36	.71	Homemaker	9	9.57	.65

*Note*. If two traits were tied in tenth place, both are presented. Alphas represent intercoder reliability for that term.

#### Data analysis strategy

Open-ended responses were analyzed and prepared in an iterative process. Following the methods employed by Bergstrom et al. (2022) and Ghavami and Peplau (2013), two trained coders first cleaned the responses for typos and spelling errors and removed any pluralization for uniformity. A frequency count for each term was prepared to record how often each term was reported by participants. Second, the reviewers standardized and combined similar root words together to remove redundancies (e.g., “terror,” “terrorist,” and “terrorism” would be combined into “terrorist”). At the third stage, short phrases were converted to a single adjective term. For example, “has negative attitudes about women” and “hates women” were converted to “misogynistic.” Once all data cleaning was completed, interrater reliabilities were computed for each term using *R* (Version 4.0.4), as indicated in Tables 2a, 2b, and 2c. Any coding discrepancies in the list of terms for each group were resolved by mutual agreement by the coders and, where necessary, the primary researcher made a final decision.

Once the list of traits was established in its final form, we investigated the core nature of each group stereotype and the degree to which intersectional identities overlapped with their religious superordinate identity. Following a similar approach employed by prior authors, we selected the 10 most commonly identified traits and explored the degree to which these traits were found in other identity groups. For example, of the 10 most common traits identified for Muslims, what proportion also appeared in the 10 most common Muslim men traits versus the 10 most common Muslim women traits? Prior studies have explored the overlap in the top 15 traits (Calabrese et al., 2018; Ghavami & Peplau, 2013; Neimann et al., 1994; Preddie & Biernat, 2020), whereas others have focused on the top 10 traits as core to a group-based stereotype (Calabrese et al., 2018; Petsko et al., 2022). For the present data, we focused on the 10 most common traits identified for each group and conducted chi-square analyses to compare the proportion of superordinate religious stereotypes that overlapped with each related intersectional stereotype. More specifically, we compared the proportion of Muslim stereotypes that overlapped with Muslim men versus Muslim women stereotypes, and we compared the proportion of Christian stereotypes that overlapped with Christian men versus Christian women stereotypes. We proceeded with chi-square analysis rather than a Fisher’s exact test (as preregistered), as all cell sizes exceeded *n* = 5 (e.g., Bower, 2003).

### Results

#### Frequency analyses

The groups varied in terms of the number of traits generated, ranging from 78 to 96 traits per group, and in terms of the specific traits identified. Traits corresponding to Muslims, Muslim men, and Muslim women are presented in Table 2a; traits corresponding to Christians, Christian women, and Christian men are presented in Table 2b; and traits corresponding to men and women are presented in Table 2c. The 10 most commonly identified traits are provided for each group, and where two traits were tied in the tenth place, both are reported. Supportive of the intersectionality hypothesis, unique traits were identified for all intersectional groups that were not found in the stereotypes of either superordinate identity. Unique traits ascribed to Muslim men included “strict,” “bearded,” and “religious outfit.” Unique traits ascribed to Muslim women included “oppressed,” “quiet,” “mistreated,” and “meek.” Unique traits for Christian women included “submissive,” “self-righteous,” and “pure.” Unique traits ascribed to Christian men included “misogynistic,” “honest,” and “brave.”

To compare how traits varied between intersectional and superordinate identities, we compared the degree of overlap between traits associated with each group. To correct for potential inflation of Type I error across six comparisons, we adjusted our threshold *p* value to .008. For Christian men, six of the top 11 traits overlapped with Christian traits, whereas two overlapped with men traits, χ^2^(1) = 3.14, *p* = .076. For Christian women, six traits overlapped with Christian stereotypes and four overlapped with women stereotypes, χ^2^(1) = 0.73, *p* = .196. Of the traits most commonly identified for Christians, six overlapped with Christian men and five overlapped with Christian women, χ^2^(1) = 0.18, *p* = .335, suggesting again that Christian stereotypes are not primarily driven by male or female representations of that group.

A different pattern was observed with Muslim intersectional stereotypes. Of the most common Muslim men traits, eight overlapped with Muslim traits and only one overlapped with men traits, χ^2^(1) = 6.77, *p* = .004. Of the top 10 Muslim women traits, three traits overlapped with Muslim stereotypes and two overlapped with women stereotypes, χ^2^(1) = 0.27, *p* = .697. Similarly, of the 11 traits most commonly identified for Muslims, eight overlapped with Muslim men and three overlapped with Muslim women, χ^2^(1) = 5.85, *p* = .007. This suggests a strong degree of overlap between Muslim and Muslim men stereotypes, more so than is observed with Muslim women and Muslim stereotypes, or Christian intersectional stereotypes.

### Discussion

Study 2 provided a conceptual replication of the main findings of Study 1. Muslim stereotypes appear strongly associated with gender and are particularly aligned with a male prototype. Christian stereotypes do not show this same degree of differentiation by gender, with less male-centred prototypes of Christianity. The results of the open-ended task identified unique stereotypes also associated with intersectional identities. Study 3 was conducted to assess whether the traits generated in Study 2 would be deemed descriptive of each respective group by a new set of participants, and whether the gendered differentiation of Muslims relative to Christians would replicate with a new sample.

## Study 3

Participants were presented with a list of the traits most commonly ascribed to each group in Study 2 and were asked to rate each for how typical that trait was of a particular group. We sought to replicate the general findings of Study 2 with respect to which traits would be deemed most descriptive of each group, including those unique to each intersectional identity. We also explored how similar each intersectional group was to their superordinate identity to determine whether Muslim men, for example, are seen as more similar to Muslims or to men. In Study 1, this was explored with visuospatial tasks, whereas in Study 3 we assessed similarity through a numeric rating scale, providing an additional metric of similarity. It was hypothesized that Christian stereotypes would be less driven by gendered assumptions and male-based prototypes. In contrast, it was expected that Muslim stereotypes would rely on male-based prototypes, with greater similarity between Muslim and Muslim men stereotypes. Further, as Muslim women are viewed as atypical of both their religious identity (as women) and of their gender identity (as Muslims), there will be greater differentiation of Muslim women from all related groups. This study was preregistered on the Open Science Framework (https://osf.io/npbj4/?view_only=f05174c28f9d4826b1d3ba18095e8e73), with an updated preregistered analysis plan found at https://osf.io/v8dkr/?view_only=b4ecebf4a10d4b7d8b9721c30cc52973.

### Method

#### Participants

An a priori power analysis was performed using G*Power (Version 3.1.9.7), with a small effect size of *f* = .10, power of 0.8, and two repeated measures, which resulted in a recommended sample of 259 participants. To offset any data loss or attrition, 339 participants were recruited from Amazon Mechanical Turk’s online participant platform in exchange for monetary compensation (US$1.95). For consistency, participants were recruited from the United States of America, with residency verified by location, latitude, and longitude. Participants were excluded from analyses due to inattentive responding (*n* = 4) or incomplete data (*n* = 26). The final sample (*n* = 308) was comprised of 158 men (51.30%) and 150 women (48.70%), with an average age of 39.56 years (*SD* = 11.43). See Table 1 for demographic characteristics.

#### Materials

##### Trait ratings

The top 10 traits identified for each group in Study 2 were compiled into a master list. Some stereotype traits were shared among the different groups, resulting in a master list comprised of 70 traits. For each group assigned to them, participants were asked, “How well do the following traits describe [group]?” Each item was rated on a 7-point scale (1 = *not at all*, 7 = *very much*), such that higher scores indicate that an item was believed to be more descriptive of that group.

##### Group similarity ratings

For each intersectional group, participants were asked to rate numerically the degree to which it was similar to each of its superordinate identities. For example, participants who had evaluated Christian women were asked, “How similar are the groups Christian women and women?” and “How similar are the groups Christian women and Christians?” Each item was answered on a 7-point scale (1 = *the groups are completely different*, 7 = *the groups are completely identical*).

#### Procedure

Participants were randomly assigned to evaluate two of the eight possible groups, such that they evaluated one intersectional group and one nonoverlapping superordinate group (e.g., Muslim men, women). Participants reviewed each group separately, completing all 70 trait ratings for one group, then 70 trait ratings for the second group. Once all trait evaluations had been made, participants provided similarity ratings for the intersectional group assigned to them. After trait ratings had been made, participants provided demographic information. Upon completion of all materials, participants were debriefed and thanked.

#### Data analytic approach

The data were explored descriptively first, examining the numeric similarity ratings made for each intersectional group, with inferential statistics comparing the perceived similarity of each intersectional identity with its superordinate identities (e.g., are Muslim men rated numerically more similar to their Muslim identity or to their gender identity?). Following this, we explored how trait ratings might vary across the eight groups evaluated. The 70 traits were condensed into several composite measures, as outlined more fully in the following lines, and a separate analysis was run for each composite trait measure. As participants evaluated more than one group, the data had a hierarchical structure, and target group differences were analyzed through a series of multilevel models with target groups nested within participants, specifying random intercepts. Multilevel models are appropriate for such hierarchically structured data, as observations are not independent of each other. This not only violates a key assumption underlying a repeated measures analysis of variance or multiple linear regression but could lead to an underestimation of standard errors and an inflated Type I error rate. Multilevel models provide a more powerful assessment of effects without inflating this error rate and avoid concerns regarding sphericity (e.g., Quene & van den Bergh, 2004). Based on the model parameters, estimated marginal means were calculated and a priori pairwise comparisons were made to determine which groups differed significantly from each other. These analyses were performed using the lme4 package (Bates et al., 2020) in R (Version 4.0.4).

### Results

#### Similarity ratings

Paired sample *t* tests were performed to compare ratings of how similar intersectional identities were perceived to be to their superordinate identities. As in Studies 1 and 2, all intersectional identities were thought to be more similar to their religious identity than to their gender identity. More specifically, Christian men were rated to be more similar to Christians (*M* = 4.97, *SD* = 1.34) than to men (*M* = 4.04, *SD* = 1.49), *t*(68) = 5.21, *p* < .001, *d* = 0.63. Christian women were rated to be more similar to Christians (*M* = 5.13, *SD* = 1.24) than to women (*M* = 4.21, *SD* = 1.59), *t*(79) = 4.57, *p* < .001, *d* = 0.51. Muslim men were rated to be more similar to Muslims (*M* = 4.77, *SD* = 1.31) than to men (*M* = 4.01, *SD* = 1.35), *t*(80) = 4.41, *p* < .001, *d* = 0.49, and Muslim women were rated to be more similar to Muslims (*M* = 4.73, *SD* = 1.47) than to women (*M* = 4.31, *SD* = 1.36), *t*(77) = 2.67, *p* = .005, *d* = 0.30. This replicates the findings of Study 1 and Study 2 that intersectional identities are considered more similar to religious superordinate identities than to gender superordinate identities, with different metrics converging on the same essential finding.

#### Trait ratings

As noted before, each target group was evaluated on a list of 70 traits that were derived from the most common traits identified in Study 2 as well as items pertaining to warmth, competence, and status (aligned with the stereotype content model; Fiske et al., 2002). To reduce Type I error and in the interests of parsimony, several composite scale items were calculated based on a priori connections between variables. A composite “warmth” scale was created by summing and averaging ratings for warm, sincere, trustworthy, nice, good natured, good, caring, helpful, loving, honest, and generous (α = .94 to .97, per target group). A “competence” scale was calculated by summing and averaging competent, competitive, confident, independent, intelligent, diligent, strong, tough, brave, and courageous (α = .84 to .92, per target group). A “socioeconomic status” scale was calculated by summing and averaging well-educated, holds prestigious jobs, and economically successful (α = .79 to .91). A “religious” scale was calculated by summing and averaging religious, faithful, holy, pure, moral, and modest (α = .64 to .89).

An “aggressive” scale was calculated by summing and averaging aggressive, angry, violent, dangerous, and mean (α = .91 to .96). A “victimized” scale was calculated by summing and averaging oppressed, submissive, meek, mistreated, weak, and quiet (α = .63 to .93). A “judgmental” scale was calculated by summing and averaging uptight, conservative, judgmental, self-righteous, arrogant, tolerant (reverse-coded), hypocritical, and prejudiced (α = .82 to .92). An “oppresses women” scale was calculated by summing and averaging controlling, domineering, strict, sexist, and misogynistic (α = .61 to .91). Finally, masculine, feminine, and terrorist were analyzed as single-item variables.

Two-level multilevel analyses were performed for each dependent variable, with target group nested within participants, specifying random intercepts. The intraclass correlation coefficient (ICC) for all dependent variables reported was above .10 (ranging .11 to .34), suggesting that a multilevel model would be appropriate for these data. Femininity and masculinity had ICC values of zero, and multilevel analyses were not performed for these variables. The results of all multilevel analyses are presented in Table 3. An *R*^2^ was computed for each multilevel model based on the formulation provided by Edwards et al. (2008). Where the analysis indicated significant differences between groups, a priori pairwise comparisons were computed. Specifically, we compared (a) how Christian men compare to Christians, (b) how Christian men compare to men, (c) how Christian women compare to Christians, (d) how Christian women compare to women, (e) how Muslim men compare to Muslims, (f) how Muslim men compare to men, (g) how Muslim women compare to Muslims, (h) how Muslim women compare to women, (i) how Christian men compare to Christian women, and (j) how Muslim men compare to Muslim women.

**Table 3.** Model parameters for multilevel analyses: Study 3.

	ICC	*F*	*df*1	*df*2	*p*	*R* ^2^
**Composite trait**						
Warmth	.22	8.95	7	301	.0001	.17
Competence	.33	11.51	7	301	.0001	.21
Socioeconomic status	.34	14.77	7	301	.0001	.26
Religious	.24	19.96	7	301	.0001	.32
Aggressive	.19	10.97	7	301	.0001	.20
Victimized	.21	28.77	7	301	.0001	.39
Judgmental	.28	9.19	7	301	.0001	.18
Oppresses women	.11	25.81	7	301	.0001	.38
Terrorist	.28	9.33	7	301	.0001	.18

We did not make every possible combination of groups to compare, as not all contrasts were of utility in this research question (e.g., comparing Muslim women to a Christian superordinate) and we hoped to minimize Type I error with added contrasts. This resulted in a total number of 10 comparisons. To offset the potential for Type I error, a Bonferroni error correction was used such that a cut-off of *p* = .005 was accepted as a statistically significant pairwise contrast. To interpret the effect size of the pairwise comparisons, an effect size was calculated that converts the *t* value to an *r*, consistent with the approach presented by Kashdan and Steger (2006). Estimated marginal means and 95% confidence intervals for all groups appear in Table 4.

**Table 4.** Estimated marginal means by group derived from multilevel analyses: Study 3.

	Christian (*n* = 79)	Muslim (*n* = 84)	Men (*n* = 83)	Women (*n* = 78)	Christian men (*n* = 78)	Christian women (*n* = 83)	Muslim men (*n* = 81)	Muslim women (*n* = 81)
Warmth	**4.92** [4.67, 5.17]	**4.46** [4.21, 4.71]	**4.36** [4.11, 4.61]	**5.11** [4.85, 5.37]	**4.92** [4.65, 5.19]	**4.93** [4.68, 5.18]	**4.11** [3.86, 4.36]	**4.69** [4.44, 4.94]
Competence	**4.38** [4.17, 4.57]	**4.70** [4.50, 4.90]	**5.12** [4.92, 5.32]	**4.67** [4.46, 4.88]	**4.78** [4.56, 4.99]	**4.32** [4.12, 4.52]	**4.70** [4.50, 4.90]	**4.15** [3.95, 4.35]
Socioeconomic status	**4.42** [4.18, 4.65]	**4.39** [4.15, 4.63]	**4.93** [4.69, 5.16]	**4.45** [4.21, 4.70]	**4.94** [4.70, 5.20]	**4.31** [4.08, 4.55]	**4.30** [4.07, 4.54]	**3.63** [3.39, 3.86]
Religious	**5.05** [4.83, 5.26]	**4.88** [4.66, 5.09]	**3.86** [3.65, 4.08]	**4.41** [4.19, 4.63]	**4.86** [4.64, 5.09]	**5.00** [4.79, 5.21]	**4.61** [4.40, 4.82]	**5.24** [5.03, 5.46]
Aggressive	**3.06** [2.74, 3.38]	**3.73** [3.41, 4.05]	**3.94** [3.62, 4.26]	**2.85** [2.52, 3.18]	**3.22** [2.88, 3.55]	**3.07** [2.76, 3.39]	**3.89** [3.57, 4.20]	**2.66** [2.34, 2.98]
Victimized	**3.12** [2.87, 3.36]	**3.40** [3.16, 3.65]	**2.82** [2.58, 3.06]	**3.90** [3.64, 4.15]	**2.98** [2.73, 3.24]	**3.54** [3.30, 3.78]	**2.90** [2.66, 3.14]	**4.53** [4.29, 4.77]
Judgmental	**4.60** [4.35, 4.86]	**4.26** [4.00, 4.52]	**4.22** [3.96, 4.48]	**3.63** [3.36, 3.90]	**4.54** [4.27, 4.82]	**4.51** [4.25, 4.77]	**4.59** [4.34, 4.85]	**3.83** [3.57, 4.09]
Oppresses women	**4.07** [3.79, 4.35]	**4.60** [4.32, 4.89]	**4.36** [4.08, 4.64]	**3.11** [2.82, 3.41]	**4.26** [3.96, 4.55]	**3.78** [3.51, 4.06]	**5.04** [4.77, 5.32]	**3.21** [2.93, 3.49]
Terrorist	**2.00** [1.65, 2.36]	**3.09** [2.73, 3.45]	**2.35** [2.00, 2.71]	**1.96** [1.59, 2.33]	**2.33** [1.95, 2.70]	**1.88** [1.53, 2.23]	**3.13** [2.78, 3.48]	**2.36** [2.00, 2.71]

*Note*. 95% confidence intervals for the estimated marginal means shown in square brackets.

##### Warmth

The model predicting warmth revealed that groups were significantly different from each other, although pairwise comparisons revealed no significant differences between groups of interest to the present discussion.

##### Competence

The model predicting competence revealed that groups differed significantly from each other. Muslim women were rated less competent than Muslim men, *t*(301) = 3.93, *p* = .0027, *r* = .22, and Muslims, *t*(301) = 3.83, *p* = .0039, *r* = .22. Muslim women trended toward being less competent than women, *t*(301) = −3.59, *p* = .009, *r* = .20. There were no gender differences for Christians.

##### Socioeconomic status

The model predicting socioeconomic status revealed that groups differed significantly from each other. Muslim women were rated lower in status than Muslims, *t*(301) = 4.50, *p* = .0003, *r* = .25; Muslim men, *t*(301) = 4.12, *p* = .0012, *r* = .23; and women, *t*(301) = 4.79, *p* = .0001, *r* = .27. Muslim men trended toward being lower in status than men generally, *t*(301) = 3.73, *p* = .0055, *r* = .21; and Christian women trended toward being lower in status than Christian men, *t*(301) = 3.72, *p* = .0057, *r* = .21.

##### Religious

The model predicting religious traits revealed a significant difference among groups. Unsurprisingly, Christian men were considered more religious than men, *t*(301) = 6.36, *p* = .0001, *r* = .34; and Christian women were considered more religious than women, *t*(301) = 3.81, *p* = .0042, *r* = .21; Muslim women were more religious than women, *t*(301) = 5.32, *p* = .0001, *r* = .29; and Muslim men were more religious than men, *t*(301) = 4.93, *p* = .0001, *r* = .27. Muslim women were considered more religious than Muslim men, *t*(301) = 4.22, *p* = .0008, *r* = .24.

#### Aggressive

The model predicting aggressiveness revealed that the groups differed significantly. Muslim women were considered less aggressive than Muslims, *t*(301) = 4.65, *p* = .0001, *r* = .26, and Muslim men, *t*(301) = 5.46, *p* = .0001, *r* = .30. Christians did not vary as a function of gender on this trait.

#### Victimized

The model predicting victimization traits revealed that the groups differed significantly from each other. Muslim women were considered more victimized than Muslims, *t*(301) = 6.46, *p* = .0001, *r* = .35, and Muslim men, *t*(301) = 9.57, *p* = .0001, *r* = .48. There were no gender differences for Christians.

#### Judgmental

The model predicting judgmental traits revealed that the groups differed significantly from each other. Christian women were considered more judgmental than women in general, *t*(301) = 4.67, *p* = .0001, *r* = .26. Muslim women were considered less judgmental than Muslim men, *t*(301) = 4.20, *p* = .0009, *r* = .24.

#### Oppressive to women

The model predicting oppression of women revealed that the groups differed significantly from each other. Muslim women were considered significantly less oppressive to women than were Muslims, *t*(301) = 6.91, *p =* .0001, *r* = .37, and Muslim men, *t*(301) = 9.26, *p* = .0001, *r =* .47. There were no gender differences for Christians.

#### Terrorism

The model predicting terrorism revealed that the groups differed significantly from each other, although none of the a priori pairwise contrasts of interest were statistically significant.

#### Exploratory models

Several additional models were run to explore the possible role of participant gender and religious identity in trait evaluations, which are reported in the online supplemental material. Participant gender did not predict ratings of any groups nor were any interaction effects between participant gender and target group detected. Participant religious identity was explored in a separate series of models, combining nonreligious participants into one group (i.e., atheists, agnostics, and nonreligious persons; 40% of the sample) and religious participants into another group (60% of the sample, almost entirely comprised of Christians). For several dependent measures, participant religious identity moderated group ratings, but there were few significant pairwise contrasts of interest. As a result, the findings presented above were collapsed across participant gender and religion. In addition, we explored the identity assumptions made by participants when making their evaluations (e.g., what ethnicity, gender, and national status did they think of for each group evaluated). These results are presented in the online supplemental material.

### Discussion

The results of Study 3 again demonstrate that intersectional identities are considered more similar to their religious superordinate identity than to their gender superordinate identity. Looking more specifically at the content of stereotypic traits, however, some clear patterns emerged. Muslim women were the most differentiated from their religious identities, differing from Muslims and Muslim men on competence, socioeconomic status, aggressiveness, victimization, oppressiveness to women, masculinity, and femininity. Muslim women were considered more religious than women and Muslim men, and less judgmental than Muslim men. Muslim men showed few significant differences from their religious and gender identities, other than the intuitive findings that Muslim men were considered more religious than men in general.

Christian groups showed few significant differences in relation to gender or religion, with Christian men, Christian women, Christians, men, and women being considered equivalent on warmth, competence, aggressiveness, victimization, and oppressiveness toward women. The few significant contrasts involving Christians were also relatively intuitive, with Christian men being considered more religious than men, Christian women being considered more religious than women, Christian women being considered more feminine than Christians, and Christian men more masculine than Christians. These results suggest that gender is a more important determinant of stereotype content for Muslim groups than for Christian groups, consistent with the findings of Studies 1 and 2.

## General Discussion

In Study 1, intersectional groups were considered conceptually closer to their religious identity than to their gender identity, and Muslims were considered conceptually closer to male versus female concepts. In Study 2, intersectional groups showed a greater degree of trait overlap with their religious superordinate identity than with their gender identity. The one exception was Muslim women, who showed little trait overlap with either their gender or religious identity. Similarly, in Study 3, Muslims and Muslim men showed no divergence from each other, whereas Muslim women were rated as significantly different from both Muslims and Muslim men on essentially all trait evaluations. This was not observed with Christians, who showed little differentiation by gender. Indeed, Christian men and Christian women rarely differed from each other or from their own Christian or gender superordinate identities. The difference in these patterns merits further consideration.

### Theoretical Implications and Possible Mechanisms

One explanation for the differentiation of Muslim women may be intersectional invisibility, wherein having multiple stigmatized identities can lead one to be overlooked, forgotten, or ignored (Purdie-Vaughns & Eibach, 2008). Although the present study did not explore invisibility directly, these results are in line with this theoretical perspective, as Muslim women are nonprototypical by way of both their gender and religious identities. That is not to say that stereotypes of Muslim women are nonexistent or undeveloped. On the contrary, very clear stereotypic representations emerged when participants were asked to evaluate Muslim women. It appears more so that one must be specifically directed to think of Muslim women when discussing Muslims, otherwise such discussions will be based on a male representation of the faith. In this way, Muslim women are rendered invisible by virtue of their intersectional identities.

This research is consistent with prior work on the intersectionality of social targets from the perceiver’s perspective. Ghavami and Peplau (2013) found that the traits ascribed to the Black superordinate identity overlapped significantly more with Black male traits than with Black female traits. This is comparable to the present results, in which the traits ascribed to Muslims align more closely with Muslim men than with Muslim women. In addition, some racial identities have been generally associated with gender in other research. Johnson et al. (2012) found that Black categories are more closely associated with male concepts, whereas Asian concepts are more closely aligned with female concepts. Similarly, Thomas et al. (2014) found that participants were slower to pair Black women (vs. Black men) with the category “Black” and slower to pair Black women with the category “women,” compared to White women. Here, it appears that Muslim religious identity is more closely aligned with male concepts than female concepts, whereas Christian religious identity is less strongly associated with one gender.

The greater gender stereotyping of Muslims relative to Christians may reflect a form of ingroup heterogeneity (Linville & Jones, 1980). Participants in this North American sample will have had greater exposure to Christians of both male and female genders, and thus have a much richer body of experiences from which to develop their group-related beliefs. For many participants, these stereotypes would be self-referential and potentially touching on family, friends, community, and colleagues. As reported more fully in the online supplement, participants did report greater familiarity and contact with Christianity compared to Islam, although such familiarity did not predict trait evaluations in multilevel analyses. It is nonetheless plausible that the greater contact and knowledge of Christians increased the incorporation of male and female conceptualizations of the Christian faith.

### Limitations and Future Directions

It should be noted that attitudes and stereotypes were assessed in this research in an acontextual format. The social context in which a group is assessed, however, can be an important determinant of attitudes. For example, different stereotypic traits may be activated when we encounter a particular group member on the street, in the workplace, at home, or in a religious setting. All may be traits associated with a particular group that become more or less salient depending on context. For example, Rosette et al. (2016) found that, in a workplace context, Black women were seen as overly dominant but not competent, whereas Asian women were seen as competent but passive. Different processes may apply to Black men in corporate America. Livingston and Pearce (2009) found that Black male CEOs (but not White CEOs) benefited from having a “babyface” in that they received a higher salary and higher prestige in their careers. The social context in which a stereotype is considered may have important implications for the additional identities attended to and the traits elicited (e.g., Petsko & Bodenhausen, 2019). Neel and Lasseter (2019) note that invisibility may not be an all or nothing status, but that invisibility may vary depending on social context. For example, stereotypes of Muslim women may be different when they are depicted as praying in a mosque, as a mother at home, as an athlete, a surgeon, or a person on the street.

The present research did not explore the importance of the perceiver’s identity in these processes. Several studies that conducted group-specific analyses found that atheists have negative stereotypes of Christians’ moral views; nonreligious people see Christians as lower in trait openness compared to non-Christians; and stereotypes of Christians’ low scientific competence are endorsed by non-Christians but not by Christians (Rios et al., 2015; Simpson & Rios, 2016). These outgroup views may become increasingly relevant as the Christian population declines in America (Pew Research Center, 2019). Future research may also assess other religious identities and additional overlapping identities (e.g., age, sexual orientation, national status). To the extent that participants were making assumptions about these identities, a systematic study may help to determine to what extent this influenced attitudes. Additionally, it would be worthwhile to assess intersectionality with other religious minority groups to determine if these results were driven by prototypicality and dominance effects or whether there is something particular to Muslims at play. Would we see similar gendered dimensions and masculine assumptions for Jewish, Amish, Buddhist, or other religious minorities relative to Christians?

Finally, it would be worthwhile to replicate this research with Muslim participants to explore whether these results are a function of participant religious identity. Although we did assess the influence of participant identification as either Christian or atheist/agnostic (see online supplement) and participant familiarity with Islam and Muslim persons, these results yielded no consistent or significant results. It should be noted that even atheists and agnostics in North America may still have much more familiarity and proximity with Christians as well as personal contact in the form of family members, coworkers, neighbours, or friends. Future research may assess whether the gendered dimensions observed for Muslims are a function of outgroup heterogeneity effects or whether they are particular to Muslims specifically. It is possible that a Muslim participant base would find Christians to be more gendered than their own religious ingroup. It is also possible, however, that Muslim participants might also perceive greater gender differences within the Muslim superordinate group compared to the Christian superordinate group, as a function of gender-segregated prayer spaces, religious practices, dress, and custom.

## Concluding Remarks

This research demonstrated through diverse methods that stereotypes of Muslims have a gendered nature. Muslim women were differentiated from Muslims and Muslim men on nearly all traits assessed, which may be attributed to their status as nonprototypical of Muslims and nonprototypical of women. Christian stereotypes showed far less gender differentiation, with a relatively even incorporation of Christian male and Christian female traits. Evidence for an intersectionality hypothesis was supported as well, with unique traits generated for each of the four intersectional groups. As such, this work contributes to our emerging knowledge of intersectionality and can contribute to a more nuanced understanding of religious stereotypes. The consequences of these group-related stereotypes and the intersectional invisibility of Muslim women remain unclear at this time, and future research would benefit from exploring the downstream effects for both perceivers and targets. The present research provides a rare systematic analysis of the landscape and structure of religious stereotypes of Christians and Muslims, work that is increasingly necessary as diversification and multiculturalism continue to grow in North America.

## Supplemental Material

sj-docx-1-gpi-10.1177_13684302221138036 – Supplemental material for The gendered nature of Muslim and Christian stereotypes in the United States Click here for additional data file. Supplemental material, sj-docx-1-gpi-10.1177_13684302221138036 for The gendered nature of Muslim and Christian stereotypes in the United States by Caroline A. Erentzen, Veronica N. Z. Bergstrom, Norman Zeng and Alison L. Chasteen in Group Processes & Intergroup Relations

## References

[bibr1-13684302221138036] Anapol A. (2018, December 8). Conservative activist disrupts campaign event for Muslim candidates. The Hill. https://thehill.com/homenews/campaign/401436-conservative-activists-disrupt-campaign-event-for-muslim-candidates

[bibr2-13684302221138036] Aron A. Aron E. N. Smollan D. (1992). Inclusion of other in the self and the structure of interpersonal closeness. Journal of Personality and Social Psychology, 63(4), 596–612. 10.1037/0022-3514.63.4.596

[bibr3-13684302221138036] Aron A. Aron E. N. Tudor M. Nelson G. (1991). Close relationships as including other in the self. Journal of Personality and Social Psychology, 60(2), 241–253. 10.1037/0022-3514.60.2.241

[bibr4-13684302221138036] Basit A. (2018). Racism, Islamophobia and Western media: An analysis how Western media portrays Muslims and Islam in the West. Muslim Perspectives, 3, 19–35.

[bibr5-13684302221138036] Bates D. Mächler M. Bolker B. Walker S. Bojesen R. H. Singmann H. Dai B. Scheipl F. Grothendieck G. Green P. Fox J. Bauer A. Krivitsky P. N. (2020). Linear mixed-effects models using “Eigen” and S4 (Version 1.1-31) [Computer software]. https://cran.r-project.org/web/packages/lme4/lme4.pdf

[bibr6-13684302221138036] Bergstrom V. N. Z. Plaks J. E. Chasteen A. L. (2022). To believe or not to believe: Stereotypes about agnostics. Psychology of Religion and Spirituality, 14(1), 21–30. 10.1037/rel0000419

[bibr7-13684302221138036] Beydoun K. A. (2017). “Muslim bans” and the (re)making of political Islamophobia. University of Illinois Law Review, 1237, 1733–1775. 10.2139/ssrn.2742857

[bibr8-13684302221138036] Bilge S. (2010). Beyond subordination vs. resistance: An intersectional approach to the agency of veiled Muslim women. Journal of Intercultural Studies, 31(1), 9–28. 10.1080/07256860903477662

[bibr9-13684302221138036] Bower K. M. (2003). When to use Fisher’s exact test. American Society for Quality, Six Sigma Forum Magazine, 2(4), 35–37. https://www.proquest.com/openview/361b2e6c2426e2fac85749a6f0c39a84/1?pq-origsite=gscholar&cbl=25781

[bibr10-13684302221138036] Bryant M. (2019, July 10). Omar hits back at “racist fool” Tucker Carlson after Fox News host’s on-air rant. The Guardian. https://www.theguardian.com/us-news/2019/jul/10/ilhan-omar-tucker-carlson-fox-news-host-racist-fool

[bibr11-13684302221138036] Cainkar L. (2004). Post 9/11 domestic policies affecting U.S. Arabs and Muslims: A brief review. Comparative Studies of South Asia, Africa, and the Middle East, 24(1), 245–248. 10.1215/1089201X-24-1-247

[bibr12-13684302221138036] Calabrese S. K. Earnshaw V. A. Magnus M. Hansen N. B. Krakower D. S. Underhill K. Mayer K. H. Kershaw T. S. Betancourt J. R. Dovidio J. F . (2018). Sexual stereotypes ascribed to Black men who have sex with men: An intersectional analysis. Archives of Sexual Behavior, 47(1), 143–165. 10.1007/s10508-016-0911-3 28224313 PMC5565715

[bibr13-13684302221138036] Chakraborti N. Zempi I. (2012). The veil under attack: Gendered dimensions of Islamophobic victimization. International Journal of Victimology, 18(3), 269–284. 10.1177/0269758012446983

[bibr14-13684302221138036] Council on American–Islamic Relations. (2018, April 23). New CAIR report: Trump’s Muslim bans increased anti-Muslim discrimination, violence. CISION PR Newswire. https://www.prnewswire.com/news-releases/new-cair-report-trumps-muslim-bans-increased-anti-muslim-discrimination-violence-300634544.html

[bibr15-13684302221138036] Crandall C. S. Bahns A. J. Warner R. Schaller M. (2011). Stereotypes as justifications of prejudice. Personality and Social Psychology Bulletin, 37(11), 1488–1498. 10.1177/0146167211411723 21659564

[bibr16-13684302221138036] Crenshaw K. (1991). Mapping the margins: Intersectionality, identity politics, and violence against women of color. Stanford Law Review, 43(6), 1241–1299. 10.2307/1229039

[bibr17-13684302221138036] Dawkins R. (2006). The god delusion. Houghton Mifflin.

[bibr18-13684302221138036] Eagly A. H. Kite M. E. (1987). Are stereotypes of nationalities applied to both women and men? Journal of Personality and Social Psychology, 53(3), 451–462. 10.1037/0022-3514.53.3.451

[bibr19-13684302221138036] Edgell P. Gerteis J. Hartmann D. (2006). Atheists as “other”: Moral boundaries and cultural membership in American society. American Sociological Review, 71(2), 211–234. 10.1177/000312240607100203

[bibr20-13684302221138036] Edwards L. J. Muller K. E. Wolfinger R. D. Qaqish B. F. Schabenberger O. (2008). A R2 statistic for fixed effects in the linear mixed model. Statistics in Medicine, 27(29), 6137–6157. 10.1002/sim.3429 18816511 PMC2587505

[bibr21-13684302221138036] Fiske S. T. Cuddy A. J. C. Glick P. Xu J. (2002). A model of (often mixed) stereotype content: Competence and warmth respectively follow from perceived status and competition. Journal of Personality and Social Psychology, 82(6), 878–902. 10.1037/0022-3514.82.6.878 12051578

[bibr22-13684302221138036] Ghavami N. Peplau L. A. (2013). An intersectional analysis of gender and ethnic stereotypes testing three hypotheses. Psychology of Women Quarterly, 37(1), 113–127. 10.1177/0361684312464203

[bibr23-13684302221138036] Gottschalk P. Greenberg G. (2008). Islamophobia: Making Muslims the enemy. Rowman & Littlefield.

[bibr24-13684302221138036] Grove R. C. Rubenstein A. Terrell H. K. (2020). Distrust persists after subverting atheist stereotypes. Group Processes & Intergroup Relations, 23(7), 1103–1127. 10.1177/1368430219874103

[bibr25-13684302221138036] Ismael T. Y. Measor J. (2003). Racism and the North American media following 11 September: The Canadian setting. Arab Studies Quarterly, 25(1-2), 101–136. https://www.jstor.org/stable/41858440

[bibr26-13684302221138036] Johnson K. L. Freeman J. B. Paulker K. (2012). Race is gendered: How covarying phenotypes and stereotypes bias sex categorization. Journal of Personality and Social Psychology, 102(1), 116–131. 10.1037/a0025335 21875229

[bibr27-13684302221138036] Kang S. K. Bodenhausen G. V. (2015). Multiple identities in social perception and interaction: Challenges and opportunities. Annual Review of Psychology, 66(1), 547–574. 10.1146/annurev-psych-010814-015025 25061671

[bibr28-13684302221138036] Kashdan T. B. Steger M. F. (2006). Expanding the typography of social anxiety: An experience-sampling assessment of positive emotions, positive events, and emotion suppression. Psychological Science, 17(2), 120–128. 10.1111/j.1467-9280.2006.01674.x 16466419

[bibr29-13684302221138036] Kteily N. Bruneau E. Waytz A. Cotterill S. (2015). The ascent of man: Theoretical and empirical evidence for blatant dehumanization. Journal of Personality and Social Psychology, 109(5), 901. https://doi.org/10/1037/pspp0000048 26121523 10.1037/pspp0000048

[bibr30-13684302221138036] Lajevardi N. Oskooii K. A. R. (2017). Old-fashioned racism, contemporary Islamophobia, and the isolation of Muslim Americans in the age of Trump. Journal of Race, Ethnicity, and Politics, 3, 112–152. 10.1017/rep.2017.37

[bibr31-13684302221138036] Linville P. W. Jones E. E. (1980). Polarized appraisals of out-group members. Journal of Personality and Social Psychology, 38(5), 689–703. 10.1037/0022-3514.38.5.689

[bibr32-13684302221138036] Livingston R. W. Pearce N. A. (2009). The teddy bear effect: Does having a baby face benefit Black chief executive officers? Psychological Science, 20(10), 1229–1236. 10.1111/j.1467-9280.2009.02431.x 19732388

[bibr33-13684302221138036] Lorde A. (1995). Age, race, class and sex: Women redefining difference. In Arthur J. Shapiro A. (Eds.), Campus wars: Multiculturalism and the politics of difference (pp. 191–198). Westview.

[bibr34-13684302221138036] Neel R. Lasseter B. (2019). The stigma of perceived irrelevance: An affordance-management theory of interpersonal invisibility. Psychological Review, 126(5), 634–659. 10.1037/rev0000143 30688473

[bibr35-13684302221138036] Neimann Y. F. Jennings L. Rozelle R. M. Baxter J. C. Sullivan E. (1994). Use of free responses and cluster analysis to determine stereotypes of eight groups. Personality and Social Psychology Bulletin, 20(4), 379–390. 10.1177/0146167294204005

[bibr36-13684302221138036] Peplau L. A. Veniegas R. C. Taylor P. L. DeBro S. C. (1999). Sociocultural perspectives on the lives of women and men. In Peplau L. A. DeBro S. C. Veniegas R. C. Taylor P. L. (Eds.), Gender, culture, and ethnicity: Current research about women and men (pp. 23–37). Mayfield.

[bibr37-13684302221138036] Perry B. (2014). Gendered Islamophobia: Hate crime against Muslim women. Social Identities, 20(1), 74–89. 10.1080/13504630.2013.864467

[bibr38-13684302221138036] Perry B. (2015). Disrupting the mantra of multiculturalism: Hate crime in Canada. American Behavioral Scientist, 59(13), 1637–1654. 10.1177/0002764215588816

[bibr39-13684302221138036] Petsko C. D. Bodenhausen G. V. (2019). Racial stereotyping of gay men: Can a minority sexual orientation erase race? Journal of Experimental Social Psychology, 83, 37–54. 10.1016/j.jesp.2019.03.002

[bibr40-13684302221138036] Petsko C. D. Rosette A. S. Bodenhausen G. V. (2022). Through the looking glass: A lens-based account of intersectional stereotyping. Journal of Personality and Social Psychology. 123(4), 763–787. 10.1037/pspi0000382 35025602

[bibr41-13684302221138036] Pew Research Center. (2019). In the U.S., decline of Christianity continues at rapid pace. https://www.pewforum.org/2019/10/17/in-u-s-decline-of-christianity-continues-at-rapid-pace

[bibr42-13684302221138036] Preddie J. P. Biernat M. (2020). More than the sum of its parts: Intersections of sexual orientation and race as they influence perceptions of group similarity and stereotype content. Sex Roles, 84(9-10), 554–573. 10.1007/s11199-020-01185-3

[bibr43-13684302221138036] Purdie-Vaughns V. Eibach R. P. (2008). Intersectional invisibility: The distinctive advantages and disadvantages of multiple subordinate-group identities. Sex Roles, 59(5-6), 377–391. 10.1007/s11199-008-9424-4

[bibr44-13684302221138036] Putnam R. Campbell D. (2010). American grace: How religion divides and unites us. Simon & Schuster.

[bibr45-13684302221138036] Quene H. van den Bergh H. (2004). On multi-level modeling of data from repeated measures designs: A tutorial. Speech Communication, 43(1-2), 103–121. 10.1016/j.specom.2004.02.004

[bibr46-13684302221138036] Reid P. T. (1984). Feminism versus minority group identity: Not for Black women only. Sex Roles, 10(3-4), 247–255. 10.1007/BF00287778

[bibr47-13684302221138036] Remedios J. D. Snyder S. H. (2015). Intersectional oppression: Multiple stigmatized identities and perceptions of invisibility, discrimination, and stereotyping. Journal of Social Issues, 74(2), 265–281. 10.1111/josi.12268

[bibr48-13684302221138036] Rios K. Cheng Z. H. Totton R. R. Shariff A. F. (2015). Negative stereotypes cause Christians to underperform in and disidentify with science. Social Psychological and Personality Science, 6(8), 959–967. 10.1177/1948550615598378

[bibr49-13684302221138036] Rosette A. S. Koval C. Z. Ma A. Livingston R. (2016). Race matters for women leaders: Intersectional effects on agentic deficiencies and penalties. The Leadership Quarterly, 27(3), 429–445. 10.1016/j.leaqua.2016.01.008

[bibr50-13684302221138036] Sides J. Gross K. (2013). Stereotypes of Muslims and support for the war on terror. The Journal of Politics, 75(3), 583–598. 10.1017/S0022381613000388

[bibr51-13684302221138036] Simpson A. Rios K. (2016). How do U.S. Christians and atheists stereotype one another’s moral values? The International Journal for the Psychology of Religion, 26(4), 320–336. 10.1080/10508619.2016.1167419

[bibr52-13684302221138036] Thomas E. L. Dovidio J. F. West T. V. (2014). Lost in the categorial shuffle: Evidence for the social non-prototypicality of Black women. Cultural Diversity and Ethnic Minority Psychology, 20(3), 370–376. 10.1037/a0035096 24730367

[bibr53-13684302221138036] Tropp L. R. Wright S. C. (2001). Ingroup identification as the inclusion of ingroup in the self. Personality and Social Psychology Bulletin, 27(5), 585–600. 10.1177/0146167201275007

[bibr54-13684302221138036] Urban L.M. Miller N. (1998). A theoretical analysis of crossed categorization effects: A meta-analysis. Journal of Personality and Social Psychology, 74(4), 894–908. 10.1037/0022-3514.74.4.894

[bibr55-13684302221138036] Willingham A. J. (2018, January 11). By 2040, Islam could be the second-largest religion in the U.S. CNN.com. https://www.cnn.com/2018/01/10/politics/muslim-population-growth-second-religious-group-trnd/index.html

[bibr56-13684302221138036] Wright S. C. Aron A. Tropp L. R. (2003). Including others (and groups) in the self: Self expansion and intergroup relations. In Forgas J. P. Williams K. D. (Eds.), The social self: Cognitive, interpersonal, and intergroup perspectives (pp. 369–390). Psychology Press.

[bibr57-13684302221138036] Zafar S. Ross E. (2015). Interreligious contact, attitudes, and stereotypes: A study of five religious groups in Canada. Canadian Journal of Behavioural Science/Revue Canadienne des Sciences du comportement, 47(1), 37–46. 10.1037/a0036720

[bibr58-13684302221138036] Zempi I. Chakraborti N. (2014). Islamophobia, victimisation and the veil. Springer.

