# Supplementary material for: The gendered nature of Muslim and Christian stereotypes in the United States
Source: Group Process Intergroup Relat. 2022 Dec 12;26(8):1726–49. doi: 10.1177/13684302221138036 (PMC10665135; doi:10.1177/13684302221138036)
Supplement: sj-docx-1-gpi-10.1177_13684302221138036 – Supplemental material for The gendered nature of Muslim and Christian stereotypes in the United States [file sj-docx-1-gpi-10.1177_13684302221138036.docx]

**Online Supplement: Additional Analyses**

**Identity Assumptions**

Perceivers often make assumptions about other non-specified social identities when thinking about social groups. When participants were asked to generate traits for Christians, for example, we do not know what gender, ethnicity, national affiliation, or other identity they might have been imagining and how that might have informed the traits they described. In Study 3, we explored the potential assumptions participants might have made about the target group’s ethnicity, immigration status, and gender. After providing trait evaluations, participants were given the following instructions: “Earlier, you were asked to provide your thoughts about [group]. When you did this, you most likely imagined a particular type of person. What was the ethnicity of the [group] person you thought of?” Participants could select the racial identity of the group member they considered, with options including White, Black, East/Southeast Asian, South Asian/Middle Eastern, Hispanic, Indigenous, or None (i.e., if they did not in fact have any assumptions). They were also asked to report the immigration status of the person they imagined: American (born in America), Immigrant to America (born overseas), Someone who does not live in America, or None. For the Christian and Muslim superordinate groups, participants were also asked to report the gender of the individual they imagined (male, female, both male and female, or none).

As seen in Table S1, participants mainly imagined the superordinate gender groups (i.e., men, women) to be White American citizens. Christians, Christian men, and Christian women were assumed to be White American citizens. In contrast, Muslims, Muslim men, and Muslim women were primarily thought to be South Asian/Middle Eastern, and immigrants or living overseas. For the superordinate category, Muslims, slightly more than half assumed a Muslim to be male, 36% imagined both male and female, only 8% imagined a female, and 5% reported making no gender assumption. For Christians, slightly less than half of participants assumed a male gender, 30% assumed a female gender, and 25% reported that they made no gender assumption. Thus, Muslims and Christians were both more likely to be thought of as male, but female-only representation was stronger for Christian stereotypes than for Muslim stereotypes, and non-gendered assumptions were also more common for Christians than for Muslims. Participants overwhelmingly reported that they imagined Christians to be American citizens (85% or higher), whereas only a small percentage imagined Muslims to be American citizens (21% or lower).

The results of the main paper suggest that Muslim stereotypes are gendered. However, it is unclear whether this is driven by perceptions of South Asian/Middle Eastern ethnicity being inherently gendered. To our knowledge, there is no research exploring gender assumptions surrounding South Asian/Middle Eastern persons, and further research is warranted to tease apart which Muslim gendered stereotypes are informed by religion and which are acting as a proxy for racial assumptions. It is also clear that participants viewed Christians to some extent as a nationality-based ingroup (American citizen) and Muslims as a nationality-based outgroup (non-Americans. Whether by exposure to Christianity or the assumption of nationality, participants may have been more likely to recognize the heterogeneity of Christians without relying on gender to inform their prototypes. Future research may seek to explore this possibility with a validated measure of contact, knowledge, and familiarity with each religious group.

**Participant Contact with Muslims as a Moderator**

Several additional measures were included in Study 3 to assess participants’ knowledge and familiarity with both Christianity and Islam. Participants were asked “Do you personally have friends, coworkers, or neighbors who are Christian?”, with responses including No, A few, or Many. Only eleven participants (3.57%) reported that they did not personally know any Christians, with most participants personally knowing a few (39.29%) or many Christians (57.14%). Participants had far less exposure to Muslim persons, with 173 (56.17%) indicating that they personally did not know a Muslim person, 122 (39.61%) reporting that they knew a few Muslims personally, and 13 (4.22%) knowing many Muslims personally. The overwhelming majority of participants reported regular contact with Christians, rendering this variable less informative for a predictive model. Rather, we explored personal contact with Muslims as coworkers, friends, and neighbors. We collapsed those who reported having a few or having many personal Muslim contacts into one group (*n* = 135, 43.83%) and those who reported no contact into another group (*n* = 173, 56.17%). Contact with Muslims was entered as a predictor into a multilevel model, including an interaction term with the target group being evaluated. As seen in Table S2, for all traits evaluated, personal contact with Muslims did not predict or moderate trait ratings. The results in the main paper are thus collapsed across personal contact.

**Participant Gender as a Moderator**

As this research explored gendered assumptions about religious groups, we explored whether participant gender predicted or moderated any of the target group evaluations. Study 3 was comprised of 158 men and 150 women, and a series of multilevel analyses were run with target group, gender, and an interaction term predicting each composite trait measure. As seen in Table S3, there were no significant effects for participant gender nor any significant interactions predicting trait ratings. The results in the main paper are thus collapsed across participant gender.

**Participant Religion as a Moderator**

We explored the potential role of the participant’s own religion on attitudes toward religious intersectional groups. Approximately half of the sample (51.30%, *n* = 158) identified as Christian or Catholic, and 8.43% (*n* = 26) of participants identified with another faith. We collapsed those of any religious affiliation into one predominantly Christian group, and collapsed all atheist and agnostic participants into another group (40.36%, *n* = 124). A series of multilevel analyses were run, entering target group, participant religious identity, and an interaction term to predict each composite trait rating. As seen in Table S4, participant religion significantly predicted trait ratings and significantly moderated group differences for each trait (with the exception of masculinity, femininity and terrorism). Due to the presence of a significant moderation, a priori contrasts were made consistent to those presented in the main paper resulting in ten comparisons. A Bonferroni error correction was applied such that a pairwise contrast would be deemed significant at p < .005. As seen in Table S5, despite the significant interaction terms, few contrasts of relevance to this discussion emerged as significant. As a result, the results presented in the main paper appear collapsed across participant religion.

| Table S1  *Identity Assumptions Reported by Participants in Study 3, as a Percentage Reporting for Each Group* | | | | | | | | | | | | | | | | | |
| --- | --- | --- | --- | --- | --- | --- | --- | --- | --- | --- | --- | --- | --- | --- | --- | --- | --- |
|  |  |  |  |  |  |  |  |  |  |  |  |  |  |  |  |  |  |
|  |  |  | Christian |  | Christian Men |  | Christian Women |  | Muslim |  | Muslim Men |  | Muslim Women |  | Women |  | Men |
| **Ethnicity** | |  |  |  |  |  |  |  |  |  |  |  |  |  |  |  |  |
|  | White |  | 79.75 |  | 85.90 |  | 83.13 |  | 9.52 |  | 2.38 |  | 7.41 |  | 61.54 |  | 60.24 |
|  | South Asian/Middle Eastern |  | 0.00 |  | 0.00 |  | 1.20 |  | 72.62 |  | 85.71 |  | 72.84 |  | 3.85 |  | 6.02 |
|  | Black |  | 8.86 |  | 11.54 |  | 6.03 |  | 8.33 |  | 1.19 |  | 4.94 |  | 11.54 |  | 3.61 |
|  | East/Southeast Asian |  | 0.00 |  | 0.00 |  | 0.00 |  | 7.14 |  | 4.76 |  | 0.00 |  | 1.28 |  | 0.00 |
|  | Hispanic |  | 0.00 |  | 1.28 |  | 2.41 |  | 0.00 |  | 0.00 |  | 0.00 |  | 0.00 |  | 3.61 |
|  | Indigenous |  | 0.00 |  | 0.00 |  | 0.00 |  | 0.00 |  | 1.19 |  | 0.00 |  | 0.00 |  | 0.00 |
|  | No assumption |  | 11.39 |  | 1.28 |  | 7.23 |  | 2.38 |  | 4.76 |  | 6.17 |  | 21.79 |  | 26.51 |
| **Immigration Status** | |  |  |  |  |  |  |  |  |  |  |  |  |  |  |  |  |
|  | American, born in America |  | 96.20 |  | 98.72 |  | 86.75 |  | 21.43 |  | 13.10 |  | 14.81 |  | 84.62 |  | 73.49 |
|  | Immigrant to America |  | 1.27 |  | 0.00 |  | 2.41 |  | 41.67 |  | 36.90 |  | 50.62 |  | 2.56 |  | 7.23 |
|  | Non-American, overseas |  | 0.00 |  | 0.00 |  | 0.00 |  | 32.14 |  | 39.29 |  | 32.10 |  | 3.85 |  | 2.41 |
|  | No assumption |  | 2.53 |  | 1.28 |  | 10.41 |  | 4.76 |  | 10.71 |  | 2.47 |  | 8.97 |  | 16.87 |
| **Gender** | |  |  |  |  |  |  |  |  |  |  |  |  |  |  |  |  |
|  | Male |  | 45.57 |  | - |  | - |  | 54.76 |  | - |  | - |  | - |  | - |
|  | Female |  | 30.38 |  | - |  | - |  | 8.33 |  | - |  | - |  | - |  | - |
|  | Both male and female |  | 0.00 |  | - |  | - |  | 4.76 |  | - |  | - |  | - |  | - |
|  | No assumption |  | 25.65 |  | - |  | - |  | 4.76 |  | - |  | - |  | - |  | - |

| Table S2  *Model Parameters for Exploratory Multilevel Analyses Exploring the Role of Contact with Muslims* | | | | | | | | | |
| --- | --- | --- | --- | --- | --- | --- | --- | --- | --- |
|  | |  | **F** |  | **df1** |  | **df2** |  | **p** |
| **Warmth** | |  |  |  |  |  |  |  |  |
|  | Group |  | 8.87 |  | 7 |  | 294 |  | .0001 |
|  | Familiarity |  | 0.00 |  | 1 |  | 306 |  | .9851 |
|  | Interaction |  | 0.39 |  | 7 |  | 294 |  | .9093 |
| **Competence** | |  |  |  |  |  |  |  |  |
|  | Group |  | 11.45 |  | 7 |  | 294 |  | .0001 |
|  | Familiarity |  | 0.14 |  | 1 |  | 306 |  | .7082 |
|  | Interaction |  | 0.59 |  | 7 |  | 294 |  | .7645 |
| **Socioeconomic** **Status** | |  |  |  |  |  |  |  |  |
|  | Group |  | 14.89 |  | 7 |  | 294 |  | .0001 |
|  | Familiarity |  | 0.10 |  | 1 |  | 306 |  | .7547 |
|  | Interaction |  | 1.88 |  | 7 |  | 294 |  | .0719 |
| **Religious** | |  |  |  |  |  |  |  |  |
|  | Group |  | 19.80 |  | 7 |  | 294 |  | .0001 |
|  | Familiarity |  | 0.14 |  | 1 |  | 306 |  | .7091 |
|  | Interaction |  | 0.54 |  | 7 |  | 294 |  | .8058 |
| **Aggressive** | |  |  |  |  |  |  |  |  |
|  | Group |  | 10.87 |  | 7 |  | 294 |  | .0001 |
|  | Familiarity |  | 11.18 |  | 1 |  | 306 |  | .0009 |
|  | Interaction |  | 0.50 |  | 7 |  | 294 |  | .8314 |
| **Victimized** | |  |  |  |  |  |  |  |  |
|  | Group |  | 29.28 |  | 7 |  | 294 |  | .0001 |
|  | Familiarity |  | 10.20 |  | 1 |  | 306 |  | .0015 |
|  | Interaction |  | 1.47 |  | 7 |  | 294 |  | .1778 |
| **Judgmental** | |  |  |  |  |  |  |  |  |
|  | Group |  | 9.24 |  | 7 |  | 294 |  | .0001 |
|  | Familiarity |  | 3.08 |  | 1 |  | 306 |  | .0801 |
|  | Interaction |  | 1.00 |  | 7 |  | 294 |  | .4325 |
| **Oppresses** **Women** | |  |  |  |  |  |  |  |  |
|  | Group |  | 25.73 |  | 7 |  | 294 |  | .0001 |
|  | Familiarity |  | 7.82 |  | 1 |  | 306 |  | .0055 |
|  | Interaction |  | 0.54 |  | 7 |  | 294 |  | .8071 |
| **Terrorist** | |  |  |  |  |  |  |  |  |
|  | Group |  | 9.40 |  | 7 |  | 294 |  | .0001 |
|  | Familiarity |  | 17.82 |  | 1 |  | 306 |  | .0001 |
|  | Interaction |  | 1.11 |  | 7 |  | 294 |  | .3561 |

| Table S3  *Model Parameters for Exploratory Multilevel Analyses Exploring the Role of Participant Gender* | | | | | | | | | |
| --- | --- | --- | --- | --- | --- | --- | --- | --- | --- |
|  | |  | **F** |  | **df1** |  | **df2** |  | **p** |
| **Warmth** | |  |  |  |  |  |  |  |  |
|  | Target Group |  | 8.86 |  | 7 |  | 294 |  | .0001 |
|  | Gender |  | 0.78 |  | 1 |  | 306 |  | .3750 |
|  | Interaction |  | 0.15 |  | 7 |  | 294 |  | .9940 |
| **Competence** | |  |  |  |  |  |  |  |  |
|  | Target Group |  | 11.49 |  | 7 |  | 294 |  | .0001 |
|  | Gender |  | 1.13 |  | 1 |  | 306 |  | .2896 |
|  | Interaction |  | 0.74 |  | 7 |  | 294 |  | .6372 |
| **Socioeconomic** **Status** | |  |  |  |  |  |  |  |  |
|  | Target Group |  | 14.96 |  | 7 |  | 294 |  | .0001 |
|  | Gender |  | 0.19 |  | 1 |  | 306 |  | .6674 |
|  | Interaction |  | 1.11 |  | 7 |  | 294 |  | .3554 |
| **Religious** | |  |  |  |  |  |  |  |  |
|  | Target Group |  | 20.11 |  | 7 |  | 294 |  | .0001 |
|  | Gender |  | 0.01 |  | 1 |  | 306 |  | .9698 |
|  | Interaction |  | 0.94 |  | 7 |  | 294 |  | .4776 |
| **Aggressive** | |  |  |  |  |  |  |  |  |
|  | Target Group |  | 11.04 |  | 7 |  | 294 |  | .0001 |
|  | Gender |  | 2.63 |  | 1 |  | 306 |  | .1061 |
|  | Interaction |  | 0.80 |  | 7 |  | 294 |  | .5897 |
| **Victimized** | |  |  |  |  |  |  |  |  |
|  | Target Group |  | 29.25 |  | 7 |  | 294 |  | .0001 |
|  | Gender |  | 3.76 |  | 1 |  | 306 |  | .0533 |
|  | Interaction |  | 1.50 |  | 7 |  | 294 |  | .1667 |
| **Judgmental** | |  |  |  |  |  |  |  |  |
|  | Target Group |  | 9.15 |  | 7 |  | 294 |  | .0001 |
|  | Gender |  | 1.47 |  | 1 |  | 306 |  | .2256 |
|  | Interaction |  | 0.51 |  | 7 |  | 294 |  | .8245 |
| **Oppresses** **Women** | |  |  |  |  |  |  |  |  |
|  | Target Group |  | 25.89 |  | 7 |  | 294 |  | .0001 |
|  | Gender |  | 0.11 |  | 1 |  | 306 |  | .7356 |
|  | Interaction |  | 0.81 |  | 7 |  | 294 |  | .5815 |
| **Terrorist** | |  |  |  |  |  |  |  |  |
|  | Target Group |  | 9.38 |  | 7 |  | 294 |  | .0001 |
|  | Gender |  | 0.20 |  | 1 |  | 306 |  | .6536 |
|  | Interaction |  | 1.08 |  | 7 |  | 294 |  | .3741 |

| Table S4  *Model Parameters for Exploratory Multilevel Analyses Exploring the Role of Participant Religion* | | | | | | | | | |
| --- | --- | --- | --- | --- | --- | --- | --- | --- | --- |
|  | |  | **F** |  | **df1** |  | **df2** |  | **p** |
| **Warmth** | |  |  |  |  |  |  |  |  |
|  | Target group |  | 9.63 |  | 7 |  | 294 |  | .0001 |
|  | Religious or not |  | 10.89 |  | 1 |  | 306 |  | .0011 |
|  | Interaction |  | 5.58 |  | 7 |  | 294 |  | .0001 |
| **Competence** | |  |  |  |  |  |  |  |  |
|  | Target group |  | 12.06 |  | 7 |  | 294 |  | .0001 |
|  | Religious or not |  | 14.97 |  | 1 |  | 306 |  | .0001 |
|  | Interaction |  | 3.79 |  | 7 |  | 294 |  | .0001 |
| **Socioeconomic** **Status** | |  |  |  |  |  |  |  |  |
|  | Target group |  | 15.09 |  | 7 |  | 294 |  | .0001 |
|  | Religious or not |  | 6.86 |  | 1 |  | 306 |  | .0093 |
|  | Interaction |  | 2.24 |  | 7 |  | 294 |  | .0310 |
| **Religious** | |  |  |  |  |  |  |  |  |
|  | Target group |  | 21.05 |  | 7 |  | 294 |  | .0001 |
|  | Religious or not |  | 5.60 |  | 1 |  | 306 |  | .0186 |
|  | Interaction |  | 3.65 |  | 7 |  | 294 |  | .0009 |
| **Aggressive** | |  |  |  |  |  |  |  |  |
|  | Target group |  | 11.63 |  | 7 |  | 294 |  | .0001 |
|  | Religious or not |  | 2.36 |  | 1 |  | 306 |  | .1252 |
|  | Interaction |  | 3.73 |  | 7 |  | 294 |  | .0007 |
| **Victimized** | |  |  |  |  |  |  |  |  |
|  | Target group |  | 29.94 |  | 7 |  | 294 |  | .0001 |
|  | Religious or not |  | 6.76 |  | 1 |  | 306 |  | .0098 |
|  | Interaction |  | 3.17 |  | 7 |  | 294 |  | .0030 |
| **Judgmental** | |  |  |  |  |  |  |  |  |
|  | Target group |  | 10.16 |  | 7 |  | 294 |  | .0001 |
|  | Religious or not |  | 2.33 |  | 1 |  | 306 |  | .1280 |
|  | Interaction |  | 7.37 |  | 7 |  | 294 |  | .0001 |
| **Oppresses** **Women** | |  |  |  |  |  |  |  |  |
|  | Target group |  | 27.03 |  | 7 |  | 294 |  | .0001 |
|  | Religious or not |  | 0.07 |  | 1 |  | 306 |  | .7877 |
|  | Interaction |  | 3.30 |  | 7 |  | 294 |  | .0021 |
| **Terrorist** | |  |  |  |  |  |  |  |  |
|  | Target group |  | 9.52 |  | 7 |  | 294 |  | .0001 |
|  | Religious or not |  | 9.90 |  | 1 |  | 306 |  | .0018 |
|  | Interaction |  | 1.40 |  | 7 |  | 294 |  | .2058 |

| Table S5  *Estimated Marginal Means by Group Derived from Multilevel Analyses (Study 3) as a Function of Participant Religious Identity* | | | | | | | | | | | | | | |
| --- | --- | --- | --- | --- | --- | --- | --- | --- | --- | --- | --- | --- | --- | --- |
|  | Muslim | | |  |  | Muslim Men | | | |  | Muslim Women | | | |
|  | Mean  Religious | Mean Non-Religious | t | p |  | Mean  Religious | Mean Non-Religious | t | p |  | Mean  Religious | Mean Non-Religious | t | p |
|  |  |  |  |  |  |  |  |  |  |  |  |  |  |  |
| **Warmth** | 4.36 | 4.59 | 0.94 | .9999 |  | 3.91 | 4.40 | 1.98 | .8372 |  | 4.81 | 4.55 | 1.00 | .9998 |
| **Competence** | 4.78 | 4.58 | 0.99 | .9999 |  | 4.64 | 4.78 | 0.71 | .9999 |  | 4.27 | 3.98 | 1.44 | .9881 |
| **Status** | 4.35 | 4.43 | 0.31 | .9999 |  | 4.22 | 4.44 | 0.92 | .9999 |  | 3.79 | 3.42 | 1.53 | .9785 |
| **Religious** | 4.78 | 5.00 | 0.99 | .9998 |  | 4.47 | 4.83 | 1.68 | .9515 |  | 5.27 | 5.23 | 0.23 | .9999 |
| **Aggressive** | 4.02 | 3.34 | 2.09 | .7704 |  | 4.36 | 3.19 | 3.62 | .0299* |  | 2.75 | 2.48 | 0.81 | .9999 |
| **Victimized** | 3.41 | 3.37 | 0.18 | .9999 |  | 2.99 | 2.76 | 0.91 | .9999 |  | 4.49 | 4.60 | 0.42 | .9999 |
| **Judgmental** | 4.38 | 4.10 | 1.09 | .9994 |  | 4.79 | 4.36 | 1.67 | .9538 |  | 3.73 | 3.93 | 0.75 | .9999 |
| **Oppresses Women** | 4.74 | 4.44 | 1.04 | .9997 |  | 5.27 | 4.74 | 1.86 | .8913 |  | 3.12 | 3.31 | 0.64 | .9999 |
| **Terrorist** | 3.41 | 2.67 | 2.04 | .7988 |  | 3.51 | 2.54 | 2.66 | .3593 |  | 2.64 | 1.91 | 1.97 | .8385 |

| Table S5 (cont’d)  Estimated Marginal Means by Group Derived from Multilevel Analyses (Study 3) as a Function of Participant Religious Identity | | | | | | | | | | | | | | |
| --- | --- | --- | --- | --- | --- | --- | --- | --- | --- | --- | --- | --- | --- | --- |
|  | Christian | | |  |  | Christian Men | | | |  | Christian Women | | | |
|  | Mean  Religious | Mean Non-Religious | t | p |  | Mean  Religious | Mean Non-Religious | t | p |  | Mean  Religious | Mean Non-Religious | t | p |
|  |  |  |  |  |  |  |  |  |  |  |  |  |  |  |
| **Warmth** | 5.41 | 4.40 | 4.08 | .0058 |  | 5.21 | 4.54 | 2.50 | .4756 |  | 5.29 | 4.39 | 3.58 | .0336 |
| **Competence** | 4.63 | 4.08 | 2.78 | .2846 |  | 5.01 | 4.46 | 2.58 | .4205 |  | 4.73 | 3.71 | 5.04 | .0001* |
| **Status** | 4.72 | 4.10 | 2.62 | .3923 |  | 5.17 | 4.65 | 2.04 | .7896 |  | 4.56 | 3.94 | 2.57 | .4246 |
| **Religious** | 5.30 | 4.79 | 2.38 | .5680 |  | 5.08 | 4.57 | 2.20 | .6944 |  | 5.14 | 4.78 | 1.65 | .9575 |
| **Aggressive** | 2.72 | 3.93 | 2.12 | .7480 |  | 3.25 | 3.13 | 0.35 | .9999 |  | 2.98 | 3.23 | 0.78 | .9999 |
| **Victimized** | 3.35 | 2.84 | 2.11 | .7545 |  | 3.39 | 2.43 | 3.69 | .0237 |  | 3.41 | 3.71 | 1.22 | .9978 |
| **Judgmental** | 4.14 | 5.11 | 3.82 | .0148* |  | 4.33 | 4.77 | 1.61 | .9660 |  | 4.10 | 5.12 | 3.98 | .0083 |
| **Oppresses Women** | 3.72 | 4.45 | 2.59 | .4095 |  | 4.08 | 4.44 | 1.20 | .9982 |  | 3.69 | 3.93 | 0.84 | .9999 |
| **Terrorist** | 1.86 | 2.11 | 0.70 | .9999 |  | 2.42 | 2.18 | 0.64 | .9999 |  | 2.09 | 1.56 | 1.38 | .9921 |

| Table S5 (cont’d)  Estimated Marginal Means by Group Derived from Multilevel Analyses (Study 3) as a Function of Participant Religious Identity | | | | | | | | | |
| --- | --- | --- | --- | --- | --- | --- | --- | --- | --- |
|  | Men | | |  |  | Women | | | |
|  | Mean Religious | Mean Non-Religious | t | p |  | Mean  Religious | Mean Non-Religious | t | p |
|  |  |  |  |  |  |  |  |  |  |
| **Warmth** | 4.61 | 3.98 | 2.52 | .4569 |  | 5.10 | 5.10 | 0.02 | .9999 |
| **Competence** | 5.18 | 5.02 | 0.82 | .9999 |  | 4.69 | 4.62 | 0.30 | .9999 |
| **Status** | 5.03 | 4.78 | 1.06 | .9996 |  | 4.50 | 4.32 | 0.67 | .9999 |
| **Religious** | 4.08 | 3.54 | 2.54 | .4477 |  | 4.55 | 4.04 | 2.12 | .7512 |
| **Aggressive** | 3.92 | 3.96 | 0.11 | .9999 |  | 3.02 | 2.55 | 1.30 | .9955 |
| **Victimized** | 3.05 | 2.47 | 2.35 | .5855 |  | 4.04 | 3.61 | 1.54 | .9775 |
| **Judgmental** | 4.17 | 4.29 | 0.49 | .9999 |  | 3.90 | 3.02 | 3.03 | .1614 |
| **Oppresses Women** | 4.26 | 4.49 | 0.82 | .9999 |  | 3.36 | 2.55 | 2.53 | .4559 |
| **Terrorist** | 2.60 | 1.99 | 1.67 | .9537 |  | 2.12 | 1.70 | 1.03 | .9997 |
